# Phospholipid Monolayers and Vesicular Membranes Show Only Minor Structural Changes After Interaction With α‐Synuclein at Low and Physiological Concentrations

**DOI:** 10.1002/cphc.70589

**Published:** 2026-10-22

**Authors:** András Major, Eva von Campenhausen, Riko Hilger, Hendrik Bruns, Titus Czajka, Diego Pontoni, Martin Rosenthal, Gerard Aguilar Pérez, Christian Hoffmann, Mohammed Al‐Azzani, Tiago F. Outeiro, Dragomir Milovanovic, Tim Salditt

**Affiliations:** ^1^ Institute for X‐Ray Physics University of Göttingen Göttingen Germany; ^2^ ESRF ‐ The European Synchrotron Grenoble France; ^3^ Department of Chemistry KU Leuven Leuven Belgium; ^4^ DZNE ‐ German Center for Neurodegenerative Diseases Berlin Germany; ^5^ Institute of Biochemistry Charité ‐ Universitätsmedizin Berlin Berlin Germany; ^6^ Department of Experimental Neurodegeneration University Medical Center Göttingen Göttingen Germany; ^7^ Algarve Biomedical Center Research Institute Faro Portugal; ^8^ Faculty of Medical Sciences Newcastle University Newcastle Upon Tyne UK; ^9^ DZNE ‐ German Center for Neurodegenerative Diseases Göttingen Germany

**Keywords:** alpha‐synuclein, GID, Langmuir monolayer, lipid vesicle, SAXS, synapsin 1, X‐ray reflectivity

## Abstract

We study how the lipid membrane structure changes upon interaction with α‐synuclein (aSyn) at low and physiological concentrations, using three different X‐ray scattering methods and two well‐established model systems, the Langmuir film balance and small unilamellar vesicles in bulk solution. While aSyn is highly membrane active, we find that at physiological protein concentration it induces only rather small changes in lipid membrane structure and vesicle shape. The vertical density profile of Langmuir lipid monolayers shows no observable changes for DPPC:DPPS and DOPC:DOPS, and moderate changes only for a model system containing gangliosides. Contrarily, the protein induces more pronounced changes in the bilayer density profile of highly curved anionic lipid vesicles, but without rupture and major remodeling of vesicular shapes as reported previously for pathological concentrations. In fact, the shape of the vesicles showed even a slight reduction in ellipticity. In addition to aSyn only, we also study the structure of the monolayer and vesicular membranes in the presence of two major synaptic proteins, aSyn and synapsin 1, at concentrations where the latter induces lipid‐protein condensates.

## Graphical abstract

The interaction of α‐synuclein with lipid membranes in the synapse is intensively discussed in view of pathologies of neurodegeneration. By X‐ray diffraction from monolayer, lipid vesicles, and synaptic vesicles at different α‐synuclein concentrations, structural changes of the membrane can be probed in response to the interaction.

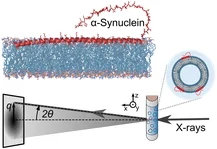

## 1 Introduction

Alpha‐synuclein (aSyn) is a small (140 aa) protein which is involved in the regulation of neurotransmitter release and synaptic vesicle cycling [1]. It is intensively researched due to its key role in the etiology of Parkinson’s disease (PD) and other synucleinopathies [1, 2]. Binding of aSyn to lipids is known to induce a conformational transition in the N‐terminal region [3] and may also induce oligomeric interactions [4, 5]. At high protein‐to‐lipid ratios and under pathological conditions, in particular for certain mutants [6], the binding of aSyn to membranes may also catalyze pathological amyloid aggregation [7].

A highly useful system to study the in vitro interaction between aSyn and lipids is the Langmuir film balance. Injection of aSyn below a pure water/air interface reduces the surface tension by 20–22 mN/m [8, 9]. When aSyn is injected under a monomolecular film of phospholipids, the pressure increases, and even more so at lower initial film pressure, indicative of aSyn penetration into the lipid membrane [10]. Indeed, combining Langmuir monolayers with fluorescence spectroscopy and circular dichroism, it was found that the first 15 amino acids of the aSyn N‐terminal segment are involved in membrane anchoring and perturbation of the hydrophobic region of the lipid monolayer, while the residues 16–30 interact only with the phospholipid polar headgroup [11]. A transition from flat‐lying helical aSyn conformation at low and at physiological aSyn concentrations in the range of 20 μM [12] to an upright conformation at elevated and potentially disease‐related concentrations was observed by vibrational sum‐frequency generation on Langmuir monolayers [13], possibly related to the promotion of amyloid fibril formation [13]. For glycosphingolipid‐containing monolayers, aSyn binding is mediated by a ganglioside‐binding domain (fragment 34–50) [14], which includes the mutation site 46 linked to a familial form of PD [15]. Alterations in the metabolism of the ganglioside GM1 have been linked to neurodegeneration: the levels of GM1 are significantly reduced in several tissues of PD patients [16], whereas in Alzheimer’s disease, increased levels of GM1 have been reported [17, 18]. In experiments using GM1‐containing lipid monolayers and vesicles as model membranes, GM1 was found to promote the fibrillogenesis and membrane disruption of Alzheimer’s disease amyloid‐β peptide [19]. Contrarily, concerning aSyn, there is compelling evidence that the binding to GM1 has a neuroprotective effect [16], possibly due to the stabilization of aSyn in a functional form [18, 20].

While many recent studies have targeted aSyn and the interaction with lipids, the primary focus was on the protein not on the lipid structure, considering conformation, aggregation, and interaction from a protein‐centric view. Contrarily, it is also of interest to study the response of lipid membrane structure and conformation upon interaction with aSyn. Already at low and physiological concentrations collective elastic response of the membrane could possibly change the lipid acyl packing, lateral ordering and the bilayer density profile ρ(z) along the membrane normal, similar to other polypeptides which interact strongly with membranes, notably antimicrobial peptides [21, 22].

In this work, we study how the lipid membrane structure changes upon interaction with aSyn at low and physiological concentrations, using X‐ray diffraction methods and two well‐established model systems, the Langmuir film balance and small unilamellar vesicles in bulk solution. The two model systems can be considered to represent the prototypic biological membrane targets of aSyn, i.e., (i) the presynaptic membrane at which exocytosis takes place and (ii) the membrane of the synaptic vesicle (SV), the organelle which releases neurotransmitters into the synaptic cleft after docking and fusion with the presynaptic membrane [23]. Before SVs are in a state ready for release upon a Ca^2+^ trigger, they are stored in the synapse in the so‐called synaptic vesicle cluster (SVC) [24]. The SVC can be considered as a condensate of vesicles and proteins, induced by the protein synapsin in a process of liquid–liquid phase separation (LLPS) [25, 26]. The SVC also recruits aSyn into the condensate, which along with synapsin is one of the most abundant proteins in the synaptic cytosol. aSyn interaction with highly curved membranes is hence of significant interest, and curvature‐sensitive binding of aSyn has been reported before in [27, 28].

Figure 1 shows a schematic of the experimental design, including a sketch of the synapse with SVC and the respective model systems. The main X‐ray scattering techniques, notably the surface‐sensitive X‐ray reflectivity (XRR) and grazing incidence diffraction (GID), as well as small‐angle X‐ray scattering (SAXS) to probe the bulk vesicle suspensions are also sketched, see Figure 1c,d,f, respectively. To illustrate a possible membrane‐bound conformation of aSyn, Figure 1b shows an extended helix structure predicted by AlphaFold (AF‐P37840‐F1) [30] and embedded in a four‐component SV‐mimetic bilayer (55 mol% DOPC, 20 mol% DOPS, 15 mol% DOPE, and 10 mol% cholesterol) using the Membrane Builder of CHARMM‐GUI [31, 32]. With the present three‐component system composed of vesicles, synapsin, and aSyn, we extend our previous work on lipid and synapsin 1 (Syn1), including the phase behavior and condensate formation in the bulk suspension probed by SAXS [33, 34], as well as the monolayer structure of the Langmuir film balance, probed by XRR and GID [35]. In the latter system, the synaptic proteins can be injected into the buffer below the film (subphase) and the subsequent interaction with the monolayer at the air–water interface can be monitored. In [35], the main observation was the following: prior binding of synapsin “protects/shields” the target membrane from structural changes when exposed to lipid vesicles (LVs) and Ca^2+^. Contrarily, in the absence of Syn1, changes in the lipid chain conformation, notably the area per lipid chain $A_{c}$ and the chain tilt angle τ, were more prominent. Here, we want to investigate whether aSyn induces a potentially stronger perturbation to the film, compared to the preserving interaction with Syn1. For concentrations in the pathological range, one might also expect conformational changes and fibrillation induced by lipid interfaces. In the synaptic context, aSyn could possibly affect adhesion/docking and/or fusion of vesicles as it has been invoked in the modulation of neuronal activity in a phosphorylation‐dependent manner [36]. Since GM1‐aSyn interactions are of significant interest here, as explained above, we have chosen to include them in the experiments.

**FIGURE 1:**
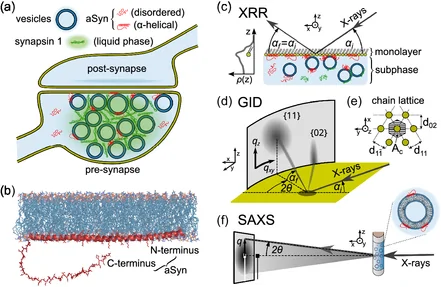
Experimental design and setup. (a) Schematic of the pre‐ and post‐synapse, highlighting the association of alpha‐synuclein (aSyn) with synaptic membranes. The presence of aSyn both at the presynaptic membrane and within the synaptic vesicle cluster (SVC) has been linked to its physiological functions [1]. Synapsin 1 (Syn1) plays a key role in SVC formation via its ability to undergo liquid–liquid phase separation (LLPS), and its interactions with other synaptic proteins, notably aSyn [29]. (b) aSyn can fold into an amphipatic α‐helix in the presence of lipid membranes, while the C‐terminal domain remains disordered [2, 3]. (c) The effects of protein binding and vesicle adhesion on the structure of planar membranes are studied using Langmuir monolayers. The lipid monolayer serves as a model of the presynaptic membrane, the synaptic proteins (aSyn, Syn1) and vesicles are sequentially added to the buffer subphase and the structural response of the monolayer is monitored using surface‐sensitive X‐ray scattering techniques. Specular X‐ray reflectivity (XRR) measures the interference of X‐rays reflected at different depths of the interface and is used to determine the average electron density profile, ρ(z), perpendicular to the monolayer plane. (d) Grazing‐incidence diffraction (GID) provides information on the lateral structure of the monolayer. The monolayer is illuminated by an X‐ray beam at a fixed, small angle of incidence ($\alpha_{i}$) to ensure total reflection. The scattered intensity is then measured as a function of two angles, 2θ and $\alpha_{f}$, corresponding to the in‐plane ($q_{xy}$) and normal‐to‐plane ($q_{z}$) components of the momentum transfer, respectively. (e) GID peaks originate from the periodicity of the acyl chain packing. Monolayers in the liquid‐condensed phase produce two peaks ({11} and {02}) corresponding to lattice spacings of a distorted hexagonal chain lattice ($d_{11}\;=\;d_{1\overset{―}{1}}$ and $d_{02}$) that can be used to determine molecular parameters such as the area per chain $A_{c}$. (f) Small‐angle X‐ray scattering (SAXS) is used to elucidate the structure of vesicles in solution with or without the synaptic proteins aSyn and Syn1.

Before presenting the main experimental results of this work, first the surface‐sensitive scattering experiments in the next section, followed by the investigation of the bulk structure by SAXS in Section 3, we want to recall the main mesoscale phenomenon of structure formation in systems of vesicles and Syn1: the formation of condensates by LLPS. As shown in Figure 2, this can be readily observed by optical microscopy. Using three different color channels, one each for the lipid, Syn1, and aSyn, we can convince ourselves that aSyn is recruited into these condensates. In contrast to Syn1, however, aSyn alone does not form micron‐scale condensates under physiological conditions [37, 38]. While aSyn can cross‐link small vesicles into “microclusters” observable by electron microscopy [39, 40], LLPS of aSyn can only be induced at pathologically high concentrations (>500 μM), at low pH, or with a high concentration of molecular crowders [41], highlighting the distinct chemical properties of Syn1 and aSyn [42]. Interestingly, the morphology of the condensates in Figure 2 looks fractal‐like. In previous work, we found that condensates undergo a concentration‐dependent transition from round domains to larger, almost percolating aggregates, with fractal appearance at high lipid‐to‐protein ratio (small P/L) and high synapsin concentration. A simple view of LLPS would let us expect rather round interfaces governed by interfacial tension between two fluid phases. Therefore, the LLPS concept for vesicle cluster formation in the context of synaptic proteins must be generalized or widened to some extent, including the possibility of more complex condensate shapes and shape transitions. This is in line with recent pipette aspiration and whole‐cell patch clamp measurements of Syn1 and aSyn condensates in cells [43]. It has been suggested that microscopic structure, notably the packing density of vesicles in the three‐component condensates, depends on the molar ratio of the two proteins [29, 37]. Using SAXS, we can test this hypothesis and measure the intervesicle distances in the SVC [33] and possibly its modulations by aSyn. Most importantly, we can test whether the vesicle structure changes when interacting with aSyn at physiological concentration. In this sense, the microscope images serve as an ideal motivation to “look into the condensates” by X‐ray diffraction, notably by solution scattering (SAXS), and to look into the structure of the lipid‐protein interface by XRR and GID.

**FIGURE 2:**
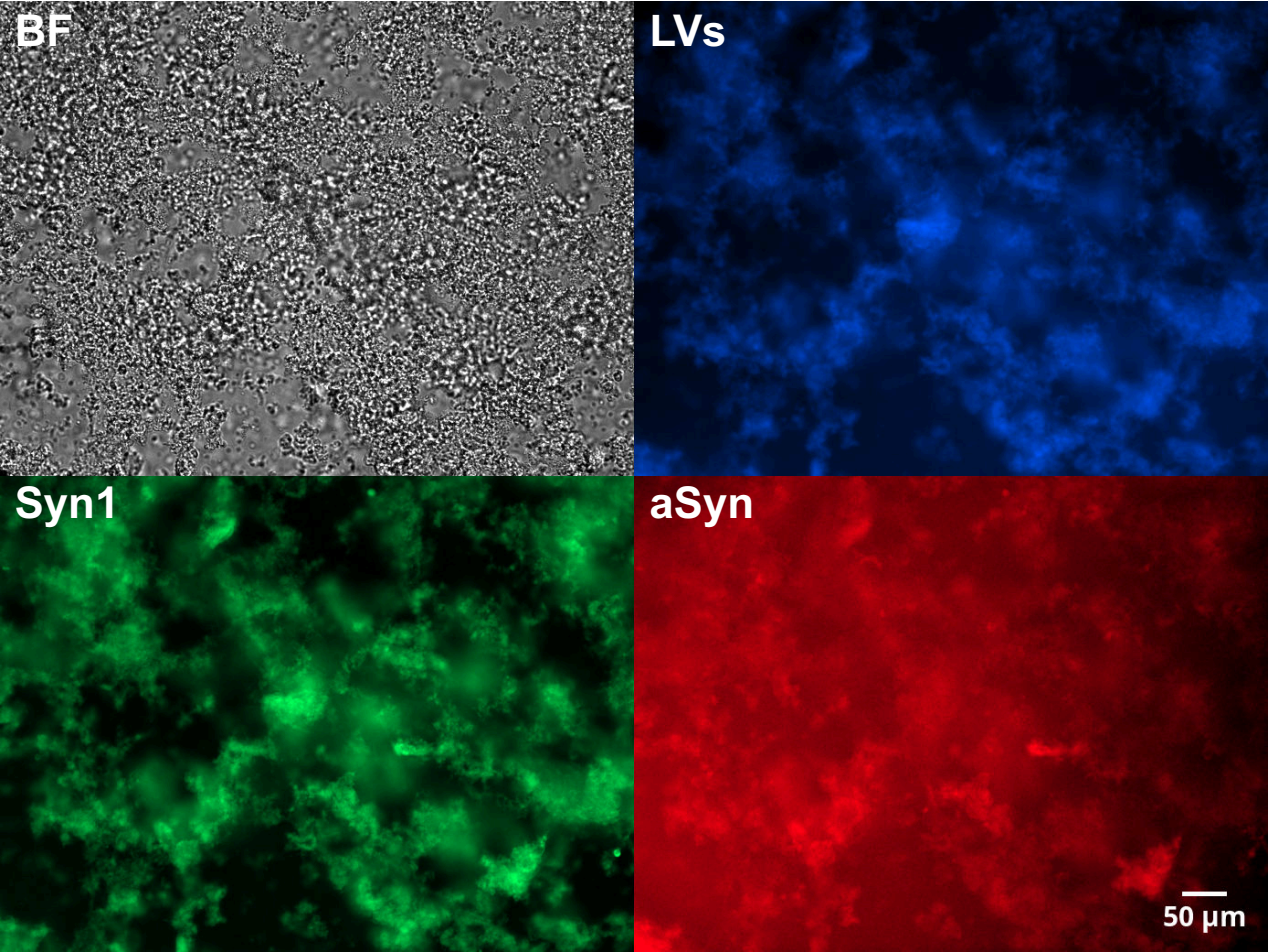
Condensate formation evidenced by optical microscopy. Small unilamellar lipid vesicles (LVs, labeled with Marina Blue DHPE, 15 mM total lipid concentration) are clustered in the presence of 6 μM EGFP‐synapsin‐1 (Syn1) and 3 μM aSyn (equimolar amounts of unlabeled and Alexa Fluor 647‐labeled). The bright field (BF) image shows microscopic vesicle‐protein condensates containing all three components visible in the fluorescence images of the same region: LVs (Marina Blue channel), Syn1 (green EGFP channel), and aSyn (red Alexa Fluor 647 channel). aSyn has a stronger homogeneous background suggesting a lower partitioning compared to Syn1 and LVs. The fractal‐like morphology of the condensates is consistent with previous observations of Syn1‐vesicle condensates (without aSyn) at high lipid‐to‐protein ratio [34].

## 2 Results and Discussion

### 2.1 Structure of Lipid Monolayers Interacting With aSyn

We used Langmuir monolayers as planar model membranes in combination with XRR and GID to elucidate structural changes in lipid arrangement upon interaction with aSyn at low (sub‐μM) concentrations. A second aim of these experiments was to investigate the modulation of vesicle–membrane adhesion by aSyn, extending our previous study involving Syn1 [35]. Here, the monolayer serves as a model of the presynaptic membrane, and the different effectors (aSyn, Syn1, vesicles, and CaCl_2_) are sequentially injected into the subphase. Throughout the Langmuir trough experiments, the buffer composition of the subphase (Tris‐buffered saline (TBS), pH 7.4) and the surface charge density of the monolayer (50 mol% negative charge) were kept constant, but we varied the lipid composition: in addition to saturated lipids (DPPC/DPPS, as in [35]), we also prepared monolayers of unsaturated lipids (DOPC/DOPS), as well as monolayers containing the ganglioside GM1 (DPPC/DPPS/GM1), which is known to specifically interact with aSyn [20].

Figure 3 shows the response of the DPPC/DPPS monolayer (1:1 molar ratio) after incubation with aSyn and after the subsequent addition of Syn1, LVs, CaCl_2,_ and EDTA into the subphase. The starting configuration was a monolayer equilibrated near a surface pressure of Π = 28 mN/m, as monitored by a Wilhelmy plate. With the motorized barrier switched off (constant monolayer area), aSyn was injected into the subphase in two steps, resulting in the surface pressure changes shown in Figure 3a. After a first injection of 1 nM aSyn (*t* = 0), the surface pressure instantly dropped by ΔΠ≃0.1 mN/m, followed by a steady increase of ΔΠ≃2 mN/m over 2.5 h. After a second injection of +9 nM aSyn (t = 150 min), resulting in a total aSyn concentration of 10 nM in the trough, the surface pressure instantly dropped by ΔΠ≃0.4 mN/m, followed by a further increase of ΔΠ≃2.5 mN/m over 2 h. Note that the reason to use two injections was rather incidental, since the aliquots of aSyn were available in two PCR tubes. Besides, it was also reasonable to monitor eventual changes already at lower concentration.

**FIGURE 3:**
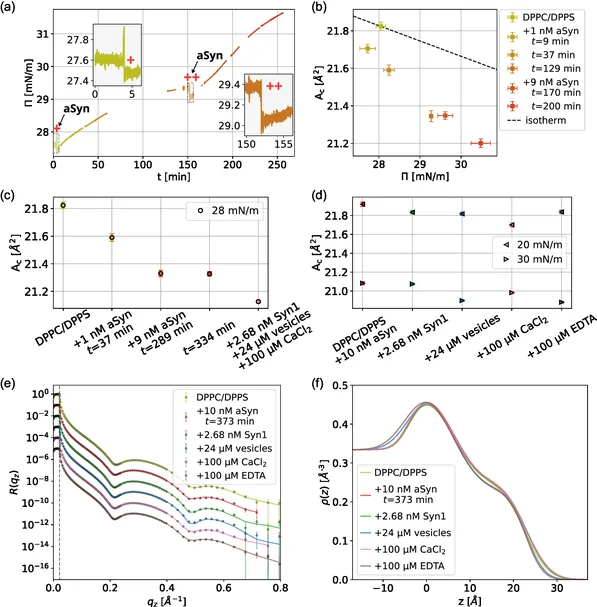
DPPC/DPPS monolayer interacting with synaptic proteins and vesicles. The starting configuration before injection of additives in the subphase was an equilibrated DPPC/DPPS (1:1) Langmuir monolayer kept close to a surface pressure of Π = 28 mN/m. (a) Increase of pressure Π(t) after aSyn injection, recorded at constant monolayer area. aSyn was injected in two steps: a first injection of 1 nM aSyn at t = 4 min (+ sign), followed by a second injection of 9 nM aSyn at t≃152 min (++ sign), resulting in a final total concentration of 10 nM aSyn in the trough. The magnified insets show the instant pressure response of the monolayer to the injections. (b) The rise in surface pressure was accompanied by a reduction of the area per lipid chain $A_{c}$, as measured by GID. This process deviates from the isotherm of the pure DPPC/DPPS monolayer indicating further compactification possibly due to a change in monolayer composition and interactions. (c) GID measurements near Π≃28 mN/m show a reduction in $A_{c}$ that reached an equilibrium between *t *= 289 and 334 min. Measurements after t = 280 min were taken at constant pressure, using the movable barrier of the trough to expand from Π≃31.5 to 28 mN/m and equilibrate at the set surface pressure. A further reduction in $A_{c}$ was observed after subsequent injections of Syn1, lipid vesicles and CaCl_2_. (d) GID data measured at Π = 30 mN/m and 20 mN/m after each injection, starting with the lipid+aSyn monolayer equilibrated for 6 h. (e) XRR data measured near Π = 30 mN/m (shifted for clarity) and least‐squares fits in log‐space to the data, along with (f) the corresponding electron density profiles ρ(z) of the monolayer for the same injection series.

The large increase in surface pressure is in line with previous studies suggesting that aSyn is highly surface active and can insert into phospholipid monolayers of various compositions [9, 10].

In addition to surface pressure measurements, structural changes of the monolayer upon incubation with aSyn were followed using GID (Figure 3b). At constant monolayer area, the increasing surface pressure was accompanied by a decreasing area per lipid chain $A_{c}$. The trajectory in Π‐$A_{c}$ space can be compared to isotherms of a pure DPPC/DPPS monolayer, measured previously by compression‐expansion cycles using the same setup, which can be approximated by a linear function within the pressure range studied here [35]. The deviation from the reference isotherm suggests that aSyn alters the interactions within the monolayer, resulting in a more compact structure than the packing of lipids alone. Electrostatic interactions could play a role in this compactification. It is worth noting that while the negative charge of DPPS facilitates the interaction with the N‐terminal domain of aSyn [2], the overall negative charge of aSyn (pI = 4.6) and especially the highly acidic C‐terminal segment could lead to electrostatic repulsion between DPPS and aSyn.

After about 4.5 h of incubation, the motorized barrier was turned back on to take X‐ray measurements at controlled surface pressure. GID measurements at Π≃28 mN/m (Figure 3c) show a reduction of the area per lipid chain after aSyn injection, which reached an equilibrium value of $A_{c}\;=\;21.33$ Å^2^ after about 5 h of incubation. Subsequent addition of Syn1, LVs and 100 μM CaCl_2_ further decreased the area per lipid chain measured at Π=28 mN/m. GID data were also taken at Π≃30 and 20 mN/m (Figure 3d) after each injection, starting with the lipid+aSyn monolayer incubated for 6 h. The additives were injected at constant area to monitor the instant pressure response (see Supporting Information), then the monolayer was first equilibrated at 30 mN/m and second at 20 mN/m using the movable barrier. The injection of Syn1 and vesicles resulted in a small reduction of $A_{c}$ at both 30 and 20 mN/m. The injection of CaCl_2_ had opposite effects on $A_{c}$ at 30 and 20 mN/m, and the addition of EDTA reversed these changes at both pressures. Overall, smaller variations in $A_{c}$ were observed after the later injections (spanning a range of approximately 0.2 Å^2^ at both 20 and 30 mN/m) than the reduction in $A_{c}$ after the addition of aSyn (approximately 0.5 Å^2^ at 28 mN/m).

We used XRR to investigate whether the observed interactions lead to measurable changes in the vertical electron density profile (EDP) of the monolayer. XRR intensity curves, $R(q_{z})$, measured near Π =30 mN/m, along with least‐squares fits in log‐space to the data are shown in Figure 3e, and the corresponding EDPs, ρ(z), are shown in Figure 3f. To keep protein consumption and radiation dose at affordable/tolerable level, every condition was measured only once. The data show that the vertical structure of the DPPC/DPPS monolayer remains unaffected by the incubation with 10 nM aSyn, i.e., there is no detectable change in the average electron density of the layers close to the surface. Note that substantial protein incorporation into the monolayer or lipid removal from the monolayer would lead to changes in the electron density and therefore result in a different reflectivity curve $R(q_{z})$. Protein insertion, in particular, would lead to shifts in position or amplitude of the modulations of the $R(q_{z})$ curve. Lipid extraction, on the other hand, would result in a decrease of modulation amplitude, ultimately approaching the monotonous reflectivity decay of a pure water surface. It therefore seems that a relatively low surface coverage (undetectable by XRR) is sufficient to induce the effects described above (changes in Π and $A_{c}$). Furthermore, the DPPC/DPPS monolayer structure remains intact and is not disrupted by aSyn. Subsequent injections only resulted in minor alterations of the XRR curves mostly confined to the high‐$q_{z}$ region (0.4 to 0.8 Å^−1^). The corresponding EDPs indicate slight changes after the injection of vesicles, CaCl_2_, and EDTA, in particular an increase of the interfacial roughness between the subphase and the lipid head group region upon vesicle injection, which may indicate an effect of vesicle adhesion or lipid exchange between the monolayer and the subphase.

While the surface activity and pressure response of the monolayer to aSyn injection are clearly evidenced by the data discussed above, the structural response is surprisingly weak, i.e., the vertical density profile and also the GID signals (aside from small shifts in the position) do not change drastically (Figure 3 and Supporting Information). It is therefore of interest to turn to a second monolayer system with potentially larger structural effects. To this end, we incorporated the ganglioside GM1, for which a specific interaction with aSyn has been reported [20]. The molar ratio of the three components (DPPC/DPPS/GM1 = 2/1/1) was chosen based on two considerations: firstly, previous Langmuir monolayer experiments of various DPPC/GM1 lipid mixtures [44] showed that DPPC and GM1 pack most tightly at a set stoichiometry of DPPC/GM1 = 3/1, suggesting the formation of DPPC/GM1 complexes defined by geometric constraints and resulting in intense GID peaks; secondly, to keep the monolayer charge density constant, we added 25 mol% DPPS (the GM1 and DPPS head groups both carry a single negative charge at neural pH). To our knowledge, we here present the first XRR and GID experiments for aSyn interacting with a Langmuir monolayer containing GM1.

Figure 4 shows the response of the DPPC/DPPS/GM1 (2:1:1) monolayer upon incubation with 10 nM aSyn. The starting configuration was a monolayer equilibrated near a surface pressure of Π = 20 mN/m, as monitored by a Wilhelmy plate. With the motorized barrier switched off, 10 nM aSyn was injected into the subphase (t = 0), and the surface pressure response was monitored at constant monolayer area (Figure 4a). After 30 min of incubation and a pressure increase of ΔΠ≃2 mN/m, the pressure was briefly reset to 20 mN/m for XRR data collection at controlled surface pressure. To keep the pressure at Π = 20 mN/m, the motorized barrier was allowed to open, increasing the monolayer area. As the monolayer did no yet reach equilibrium, the motorized barrier was switched off again at t≃63 min and the surface pressure was monitored at constant monolayer area, resulting in a further increase by ΔΠ≃6.5 mN/m over 3.5 h. Due to the increase in monolayer area between t≃30 and 63 min, the total pressure increase (ΔΠ≃8.5mN/m) was reduced compared to the constant‐area condition; however, this value is still comparable to the pressure increase observed in a control experiment conducted at constant area (ΔΠ≃9 mN/m over 3 h after injection of 10 nM aSyn at $\Pi_{i}≃19\;\mathrm{mN}/m$, see Figure S13b. GID scans were recorded to probe changes in lipid packing at constant monolayer area following the injection of aSyn (Figure 4b). Similarly to the DPPC/DPPS monolayer (Figure 3b), the surface pressure increase is accompanied by a decrease in the area per lipid chain $A_{c}$. The deviation from the reference isotherm of the pure lipid monolayer suggests that aSyn alters the interactions within the monolayer, resulting in a more compact structure than the packing of lipids alone. XRR curves were recorded at both Π= 20 and Π = 26mN/m to monitor the changes in the vertical density profile of the monolayer. The XRR intensity curves $R(q_{z})$ with least‐squares fits in log‐space to the data are shown in Figure 4c,d, and the corresponding EDPs ρ(z) are shown in Figure 4e,f.

**FIGURE 4:**
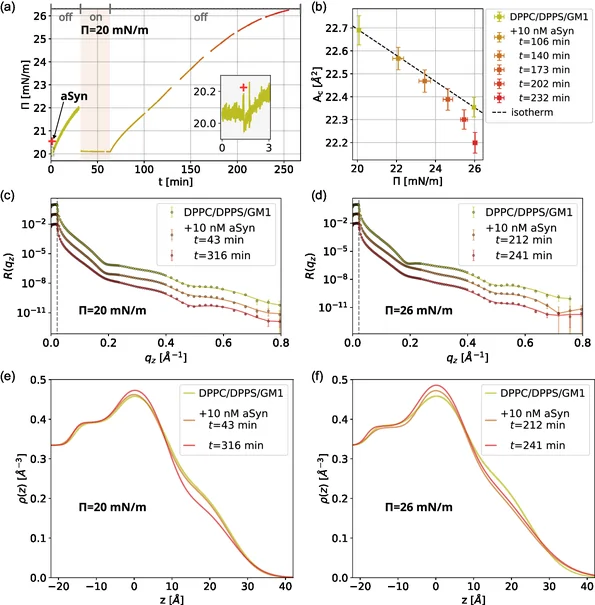
GM1 promotes the insertion of aSyn in a lipid monolayer. The starting configuration was an equilibrated DPPC‐DPPS‐GM1 (2:1:1) Langmuir monolayer kept close to a surface pressure of Π = 20 mN/m by the movable barrier of the trough. (a) Increase of pressure Π(t) after injection of 10 nM aSyn (+ sign). The motorized barrier (pressure control) was turned off during the first and third part of this experiment to follow the pressure increase at constant monolayer area, whereas between t≃30 and 63 min (orange shaded area) the pressure was set to Π = 20 mN/m for X‐ray data collection at constant pressure. During this time, the barrier continued to open, thereby increasing the monolayer area until we switched back to constant‐area mode at t≃63 min. (b) Area per lipid chain measured by GID after the addition of 10 nM aSyn. The deviation from the lipid monolayer isotherm indicates further compactification possibly due to a change in monolayer composition and interactions. (c) XRR data collected at Π = 20 mN/m and (d) at Π = 26 mN/m before/after injection of 10 nM aSyn and least‐squares fits in log‐space to the data (shifted for clarity), along with the corresponding electron density profiles ρ(z) shown in (e) and (f), respectively. Following a long incubation time (t>200 min), a change in the XRR curves is observed, giving rise to an increased electron density at the head group region and a decreased electron density at the tail group region, which is consistent with the insertion of aSyn at the depth of the lipid head groups. Note that the additional layer in ρ(z) around z≈−10Å below the head group maximum (z≈0) is attributed to the glycans attached to the GM1 head groups.

The effect of including 25 mol% GM1 in the monolayer is evident in the XRR curves, which differ from the XRR curves of DPPC/DPPS (Figure 3e): the first maximum between $q_{z}≃0.2$ and 0.4 Å^−1^ is flattened and instead the curve has a shallow minimum around $q_{z}≃\;0.3$ Å^−1^, in line with previous XRR measurements on similar monolayers [45, 46]. To accurately fit the XRR curves in the range of $q_{z}≃0.2$ to 0.4 Å^−1^, a 3‐slab model of the vertical EDP was used, including an additional layer representing the branched oligosaccharide (glycan) chains attached to the GM1 head group. The measured thickness of the glycan layer ($L_{\mathrm{glycan}}≃12$ Å) is consistent with previous X‐ray diffraction analysis of PC + GM1 bilayers containing 20–30 mol% GM1 [47], indicating that the oligosaccharide portion of the GM1 head group is nearly fully extended from the monolayer towards the subphase. In striking contrast to the DPPC/DPPS monolayer, the addition of aSyn to the DPPC/DPPS/GM1 monolayer has an effect on the XRR curves not only at high $q_{z}$, but also at low $q_{z}$ ($q_{z}<0.4$ Å^−1^). The XRR curve measured t = 43 min after the injection of aSyn is still similar to the XRR signal from the pure monolayer (Figure 4c). After an extended incubation time (t>200 min), the shape of the XRR curve changes, with a flattening of the first minimum near $q_{z}≃\;0.2\;$Å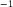 and a steeper decrease in reflected intensity between $q_{z}≃0.2$ and 0.4 Å^−1^. The corresponding density profiles ρ(z) (Figure 4e,f) indicate an increased electron density in the head group region and a decreased electron density in the tail group region. This is consistent with aSyn insertion in the head group region: at constant surface pressure, the insertion of aSyn may dilute the lipid packing and reduce the apparent electron density in the hydrocarbon tail region, while adding scattering material in the head group region.

One major limitation of the Langmuir trough is the fact that lipid systems which exhibit only short range order at room temperature in a bilayer (L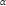 phase), exhibit higher liquid–crystalline order when prepared as a monolayer, as evidenced from the GID peaks of the acyl chain packing. While this effect is heavily exploited for structural changes as a sensitive readout, for example after protein adsorption, it is also quite artificial, since the bilayer membrane has an equivalent to these signals only in the gel phase.

It is therefore of interest to also probe monolayers in a pure liquid‐disordered state without long‐range crystalline GID peaks but instead the typical GID signal of fluid bilayers. To this end, we next turn to a monolayer system with only unsaturated lipid chains composed of DOPC/DOPS in 1:1 molar ratio.

In contrast to the strong GID peaks originating from the long‐range order within liquid‐condensed domains of saturated lipids, the GID signal from unsaturated monolayers is weaker and requires high surface pressures for acceptable signal‐to‐noise ratio. Therefore, we performed our measurements at a relatively high surface pressure of Π=35 mN/m.

Figure 5 shows the vertical structure of the DOPC/DOPS monolayer (1:1 molar ratio) as measured by XRR. Similarly to the DPPC/DPPS monolayer, the XRR signal from the DOPC/DOPS monolayer was largely unchanged upon incubation with aSyn. XRR curves measured after the subsequent addition of Syn1, LVs, CaCl_2_, and EDTA only show slight variations in the last few noisy data points ($q_{z}>0.6$ Å^−1^) and the EDP remained entirely unchanged. This may be partly due to the high surface pressure (Π=35 mN/m) that was necessary to have good signal from the unsaturated lipids with fluid chains, but may have prevented the insertion of proteins. However, the lack of change upon vesicle and calcium addition might suggest a shielding effect by the proteins, as proposed earlier for Syn1 [35].

**FIGURE 5:**
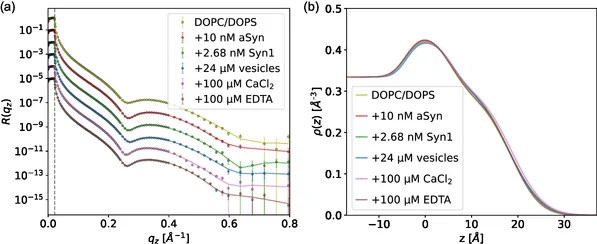
DOPC/DOPS monolayer interacting with synaptic proteins and vesicles. The starting configuration before injection of additives in the subphase is an equilibrated DOPC/DOPS (1:1) Langmuir monolayer kept close to a surface pressure of Π=35 mN/m by the movable barrier of the trough. (a) XRR data collected at Π = 35 mN/m (shifted for clarity) and least‐squares fits in log‐space to the data, along with (b) the corresponding electron density profiles, ρ(z).

GID data from the unsaturated DOPC/DOPS monolayer showed the fluid acyl chain correlation peak (see Supporting Information) with its maximum close to $q_{z}\;=\;0$ axis at around $q_{xy}≃1.4$ Å^−1^ as known for fluid lipid bilayers [22, 48], as opposed to the 2D crystalline order of the saturated lipids resulting in two ({11}, {02}) lattice peaks. This clearly indicates that the unsaturated lipids do not pack in crystalline phases. To this end, the DOPC/DOPS monolayer is a more realistic model system. Here as well, no strong structural changes are observed after injection of aSyn.

### 2.2 SAXS Analysis of Vesicle‐Protein Co‐Structures

#### 2.2.1 Structure of LVs Interacting With aSyn

Figure 6 shows the SAXS results for the aSyn‐LV suspensions. The one‐dimensional intensity curves I(q) are shown after azimuthal averaging of the radially symmetric scattering patterns, as a function of the momentum transfer q (modulus of the scattering vector). Interestingly, the SAXS curves, shifted vertically for clarity, exhibit only minor changes in presence of aSyn (Figure 6a). Previous SAXS studies reported major structural changes of LVs upon the addition of aSyn at pathologically high concentrations (70–270 μM) [49], at which aSyn was found to strongly interact with LVs of high negative surface charge density (100 mol% DLPS or DMPS), resulting in a break‐up into small and mostly disc‐ or rod‐like lipid‐protein particles. In contrast to Galvagnion et al., we here study relatively low bulk concentrations of aSyn from 0 to 25 μM, close to the physiological bulk concentration in the synapse of ca. 20 μM [12]. Further, we work with a LV composition mimicking the lipid composition of synaptic vesicles (SVs) [25, 50]. To analyze the acquired data, we have implemented and fitted several structural models to the data, all based on parameterizing the radial EDP of the vesicle lipid bilayer as a sum of three Gaussians representing the headgroup maxima and the midplane minimum [51, 52],

**FIGURE 6:**
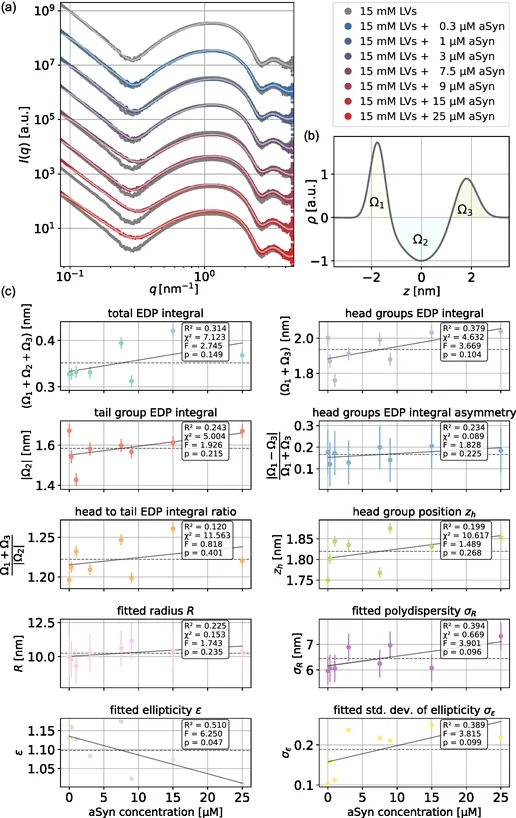
Structure of lipid vesicles in the presence of aSyn. (a) SAXS data and fits for 15 mM LVs at increasing concentrations of aSyn (shifted for clarity, data colored in blue to red, least‐squares fit in light gray). The SAXS curve of the 15 mM LV solution without aSyn (dark gray) is plotted below each LVs+aSyn sample as a reference. (b) Illustration of the parameters derived from the electron density profiles (EDPs) corresponding to the least‐squares fits in (a). The EDP of the 15 mM LVs (no protein) is shown as a reference. $\Omega_{1,2,3}$ are the EDP integrals of the inner head group, the central tail groups and the outer head group, respectively. (c) Different parameters of fits with the ellipsoidal asym. model depending on the aSyn concentration from 0 to 25 μM. $R^{2}$ values, F‐statistic of the linear with respect to a constant model, and corresponding *p*‐values are given for each parameter in the legend.

(1) $$\rho (z)=\sum_{i\;=\;1}^{3}\rho_{i}\exp[-\frac{{\left(z-z_{i}\right)}^{2}}{2\sigma_{i}^{2}}]$$

with the peak amplitudes $\rho_{i}$, peak positions $z_{i}$, and the Gaussian widths $\sigma_{i}$. For all fits, $z_{1}=z_{3}\equiv z_{h}$ and $z_{2}\equiv 0$, i.e., the head group distances from the bilayer midplane were set equal for the two leaflets. Since only the ratios, not the absolute values of the amplitudes $\rho_{i}$ determine the scattering curve (up to a scaling of intensity), we set $\rho_{2}\equiv -1$. Both symmetric EDPs (sym.) with $\rho_{1}=\rho_{3},\sigma_{1}=\sigma_{3}$ and asymmetric EDPs (asym.) with $\rho_{1}\neq \rho_{3},\sigma_{1}\neq \sigma_{3}$ were tested. On larger length scales, we have attributed either a spherical or an ellipsoidal shape to the LVs. In the spherical vesicle model, aside the EDP parameters, the radius R and the polydispersity $\sigma_{R}$ enter as two additional fit parameters. In the ellipsoidal model, which contains the spherical model as a special case, the LVs are represented as rotational ellipsoids (spheroids) with two equal axes R and one axis ε⋅R, where ε is the ellipticity with an ellipsoidal polydispersity $\sigma_{\varepsilon }$ for the corresponding width of the ensemble distribution function. Both for the spherical and the ellipsoidal polydispersity we assume Gaussian normal distributions. All model functions are detailed in the Supporting Information. As we expect aSyn to bind to the outer leaflet of the vesicle bilayer, we have also redesigned the LV models by including an additional Gaussian in the EDP to account for a layer of adhered protein. However, these attempts did not yield consistent and meaningful fit results, indicating overparameterization and/or an insufficient signal of such a layer, for example due to a partial surface coverage. For this reason, we deliberately keep the model at a lower level of complexity, accepting that changes in the outer headgroup density may reflect both direct and indirect contributions of the protein. In other words, in an effective manner, structural changes in the SAXS curve resulting from protein adhesion both by direct contributions of the amino acids interacting with the bilayer as well as the collective response of the lipids could show up in the simple EDP model as an increase of the outer headgroup maximum. Accordingly, we base the analysis on the so far best performing models: the spherical and ellipsoidal models with asymmetric EDP. Keeping the simpler model also for the LVs with added proteins is also justified by the fact that the scattering curves only show very minor changes in presence of aSyn, see Figure 6a, showing the concentration‐dependent SAXS data along with the respective fitting curves using the ellipsoidal vesicle model with asymmetric EDP. This model convincingly captures all important features of the SAXS data, except for the low‐q region, possibly due to the presence of larger particles (aggregates) which would manifest themselves in this q‐range.

Already when fitting SAXS data of pure LVs without protein, we experienced quite some ambiguity, i.e., different vesicle EDPs can describe the data almost equally well. By varying the initial guess and the bounds of the fitting algorithm, a number of equally precise fits with significantly different EDPs can be obtained. This ambiguity can be attributed to a certain overparameterization. It can also be rationalized when considering the resolution limit resulting from the q‐value, where the background exceeds the vesicle signal. However, we noted that while the individual parameters $\rho_{1,3}$ and $\sigma_{1,2,3}$ individually are ambiguous, the areas of the three EDP Gaussians show consistency among numerous fits of the same curve. For this reason, we introduce the quantities $\Omega_{1,2,3}$ for the characterization of the vesicle EDP under influence of aSyn. As illustrated in Figure 6b, $\Omega_{1,2,3}$ are defined as the integrated EDP areas of the inner head group, the central tail groups and the outer head group respectively. In this way, the parameters describing the structure of the EDP are effectively reduced by a factor of two. By placing $\Omega_{1,2,3}$ in different relations, we aim to obtain reliable measures of relevant properties such as the total EDP integral, the EDP asymmetry and the ratio of head groups to tail groups.

Figure 6c shows structural parameters derived from the fitted EDPs as functions of the aSyn concentration. To estimate the error of every quantity, we calculate the standard deviation over a number of fits carried out with different initialization and meta‐parameters. Note that the statistical errors of each individual fit due to the propagated errors of photon noise are well below the systematic variation when changing the meta parameters of preprocessing or least‐square fitting. To estimate the effective errors for the fit parameters, we vary parameter initialization, parameter bounds, and a scaling constant involved in background subtraction. The latter accounts for systematic errors due to (uncontrolled) thickness variations between sample and background quartz capillaries, see Figures S17 and S18. For the batch fitting and averaging, the approximate analytical form of the spherical asym. model according to Brzustowicz and Brunger [51] is particularly advantageous (see Equation (S8)), minimizing the amount of computation power. Contrarily, the ellipsoidal asym. model involving numerical integrals is impractical for this approach. For these reasons, the errors of the spherical model are also transferred to the data points of the more general ellipsoidal asym. model shown in Figure 6b. For each scattering curve, the fit parameter space was explored with 5193 starting points, leading in total to 3–11 equally precise fits per sample with an average parameter novelty of at least 10%. The resulting effective errors seem reasonable. The total EDP integral shows only a relatively small spread, i.e., is robust with respect to the variation of the meta parameters of the fit. Contrarily, the EDP asymmetry parameter exhibits a larger dependance, resulting in correspondingly larger error margins. Having described the fit procedure and strategy to estimate the fit errors, we now address the structural changes of the vesicular bilayer following the interaction with aSyn.

The data in Figure 6c is fitted by linear regression in order to capture dependance of the structural parameters on aSyn concentration. The simple linear model is justified since the effects of aSyn are small, and hence only the lowest order should be kept, also in view of avoiding overfitting. The general trend is as follows: there is an increase in total scattering density (total EDP integral) and membrane asymmetry, as expected for protein adhesion. At the same time, shift of the size distribution, i.e., the radius and the polydispersity both increase by about 1 nm, which can be observed over the covered concentration range. Further, a decrease in ellipticity along with an increase in the ellipsoidal polydispersity $\sigma_{\varepsilon }$. All of these observations seem plausible consequences of protein interaction and adsorption. We should note, however, that the $R^{2}$‐value indicates that a significant fraction of the variance is not explained by the fit, related to remaining deficits and ambiguities of the analysis, typical for cases when the effects are near the limit of robust signals. At the same time, the estimate for the systematic errors of the individual curves is not completely satisfactory. Importantly, testing the linear regression with respect to a constant model by a F‐statistics, the resulting *p*‐values (see labels in Figure 6c) exceed the threshold of $p^{*}\;=\;0.05$ commonly associated with statistical significance, for all parameters, but the ellipticity ε, which we can hence consider as a main parameter accounting for the systematic increase of intensity at the position of the first minimum. Within the statistical margins associated with ambiguity of the fits, we hence have rather unperturbed EDPs. Importantly, all curves can be fitted with the same vesicle model and little systematic change in the EDPs. We can conclude that aSyn has only minor effects on the overall structure of LVs at the examined concentrations (0 to 25 μM) and that the structural integrity of the vesicles is preserved. Note that at the same time, the membrane affinity of aSyn [53] predicts a substantial fraction of membrane “binding sites” to be occupied under our experimental conditions, as detailed in the Supporting Information.

Prompted by the “null” result and the discrepancy with respect to the report by Galvagnion et al., we have carried out a few additional SAXS measurements at higher aSyn concentration, and making use of beamtime at the dedicated SAXS instrument of beamline BM29 of ESRF [54], offering a wider q‐range and a better background subtraction based on a flow‐through capillary. The results are shown in Figure 7, for 15 mM LVs with 0 μM (pure lipid) and 75 μM aSyn, corroborating the findings at lower aSyn concentration, namely, a preserved vesicular shape, fitted here to the ellipsoidal vesicle model with asymmetric EDP. Further, the EDPs of both the full‐q range fit to the ellipsoidal model and the flat bilayer model (for $q\geq 0.7\;{\mathrm{nm}}^{-1}$) are rather consistent. The main change at high aSyn concentration is an increase in the EDP asymmetry.

**FIGURE 7:**
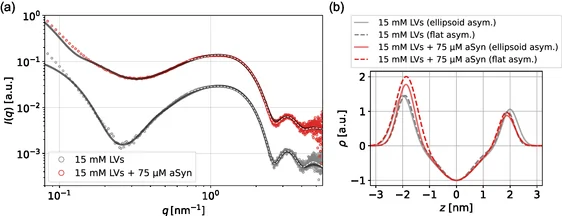
SAXS data of 15 mM LVs with 0 or 75 μM aSyn, shifted for clarity. The ellipsoidal (solid line) and the flat model (dashed line), both with asymmetric EDP were fitted to the data. The flat bilayer model is only valid for high‐q data, here fitted for $q\geq 0.7\;{\mathrm{nm}}^{-1}$.

#### 2.2.2 Structure of Vesicles Interacting With aSyn and Syn1

Next, we extend the SAXS investigation to samples containing both aSyn and Syn1. With respect to the finding presented above, namely, the mild structural changes induced by aSyn, we may ask: does this picture change in the presence of Syn1 as one of the most abundant synaptic proteins, and in particular as the effector protein for formation of the SVC? Knowing that Syn1 induces condensates via LLPS with strong intervesicle adhesion in the LV4 model [33, 34], do these “bound vesicles” in the condensates interact differently with aSyn? Conversely, how does aSyn modulate the vesicle structure in the Syn1‐induced condensates? Since cryo‐EM showed that the Syn1 induces strong adhesion of LVs with flattened adhesion sites, the scattering function of a condensate cannot be described as a product of form and structure factor. Contrarily, for SVs which are more robust in shape, this picture can still be approximately used [33]. We therefore refrain from modeling shape changes of the vesicles and instead concentrate only on the bilayer EDP.

Figure 8 presents the SAXS data and fitting results for the aSyn‐Syn1‐LV suspensions and some first (pilot) data for aSyn‐Syn1‐SV. For LV, we focus on the bilayer form factor, without any attempt to model the intervesicle correlations and shape changes which manifest themselves in the small‐q region (see Figure S15). We therefore limit our analysis to the high‐q region $q>0.7\;{\mathrm{nm}}^{-1}$, which is dominated mainly by the length scales of the lipid bilayer. Accordingly, we describe this q‐range with a model function for a flat bilayer with asymmetric EDP (see Supporting Information). The scattering curves and least‐square fits for different concentrations of aSyn and Syn1 are shown in Figure 8a. The respective EDPs in Figure 8b show that the basic bilayer structure remains intact. The resulting EDP parameters are detailed and plotted in the Supporting Information. While addition of Syn1 shows notable effects in all six parameters of the EDP, the effects of aSyn are ambiguous or small. As a consequence of increasing Syn1 concentration, the total EDP integral as well as the head group integral is growing. However, not only the amount of scattering material increases but also the EDP asymmetry and the ratio of head groups to tail groups. We attribute these effects to protein adhesion and possibly insertion into the vesicle lipid bilayer. Since Syn1 (705 aa) is significantly larger than aSyn (140 aa), it is plausible that adhesion/insertion of Syn1 has larger effects on the vesicle scattering curve.

**FIGURE 8:**
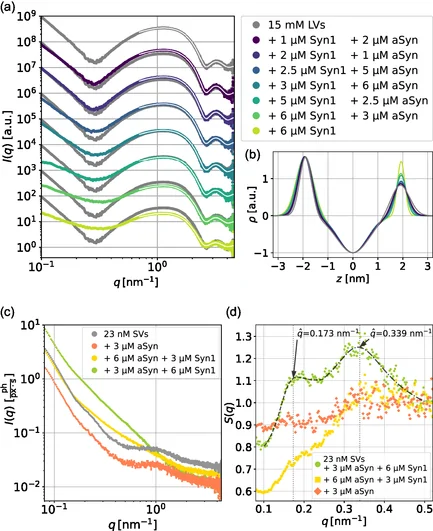
Structure of vesicles in the presence of both aSyn and Syn1. (a) SAXS data for LV and fits (white) in the region $q>0.7\;{\mathrm{nm}}^{-1}$, shifted for clarity. As a reference, the SAXS curve of the LV solution without protein is plotted together with each curve. (b) EDPs corresponding to the least‐square fits. (c) SAXS curves for native synaptic vesicles (SVs) with or without the synaptic proteins aSyn and Syn1. (d) Corresponding structure factors S(q) obtained by dividing the SAXS intensity of the SV‐protein samples by the SAXS intensity of the pure SV suspension (shifted vertically for clarity). The S(q) of the sample with the highest Syn1 concentration shows 2 peaks, the peak positions $\hat{q}$ were determined by fitting a Lorentzian with linear background to the data.

Finally, Figure 8c,d shows first data towards the structure of SVs interacting with aSyn and/or Syn1. Note that SVs possess a dense protein corona, which distinguishes them from synthetic LVs. This important difference is reflected in the SAXS curves, which exhibit significant qualitative differences compared with the SAXS curves of LVs. Since Syn1 forms a liquid phase with SVs [34], the structure factor S(q) cannot be set to 1 as for monodisperse vesicle solutions. Mathematical models of S(q) have previously provided evidence that SV‐Syn1 condensates have a loosely bound, noncompact structure [33]. Here, we approximate the structure factor as $S(q)\;\approx \;I_{\mathrm{SV}+\mathrm{protein}}(q)/I_{\mathrm{SV}\;\mathrm{pure}}(q)$, assuming that the SV form factor is roughly the same in both cases. The structure factors are shown in Figure 8d The curve for SV and aSyn only (in the absence of Syn1) shows the weakest modulation, indicative of an ideal solution (S(q)≃1). The maximum at 0.35 nm^−1^, which appears in all three depicted curves can be tentatively attributed to changes in the form factor when proteins adsorb to the SV surface, which, however, is not related to the condensate structure factor of interest here. Rather, we must focus on the low‐q region, where all three curves behave very differently. While the sample with 6 μM Syn1 features a pronounced shoulder at $\hat{q\;}=\;0.173\;{\mathrm{nm}}^{-1}$, this shoulder is smaller in the sample with 3 μM Syn1 and it is completely absent in the sample without any Syn1. Based on the different behavior of this curve feature among the samples, we interpret it as a structure factor maximum. We obtain an intervesicle distance of roughly $d=2\pi /\hat{q}\approx 34.15\;\mathrm{nm}$ within the compact fluid model. Taking into consideration that a radius of ca. $R_{\mathrm{SV}}\approx 27\;\mathrm{nm}$ has been determined earlier for these purified SVs, we note that $d<2R_{\mathrm{SV}}$. This perfectly matches the observations of Neuhaus et al. [33] in clusters of SVs and Syn1. Since such a tight spacing is implausible with respect to the SV architecture and also EM evidence, Neuhaus et al. have argued for a noncompact cluster structure and supported this hypothesis with simulations of clusters. Here, we could extend this analysis to clusters of SVs, Syn1, and aSyn. However, as the current quality of structure factors is limited and we do not see an effect of different aSyn concentrations on the $\hat{q}$‐position, we will leave an in‐depth analysis to future projects with cleaner structure factors and a wider range of Syn1 to aSyn ratios.

## 3 Conclusions

We conclude as follows: aSyn is highly membrane active (as shown previously) but does not disrupt the membrane structure, at least not in the lipid model systems and in the range of physiological protein concentrations studied here. This is in contrast to the experiments by the group of C. Galvagnion, who report membrane disruption and/or shape transformations of vesicles at high and potentially pathological aSyn concentrations. In the Langmuir film balance, the surface pressure instantly dropped by 0.1–0.5 mN/m (depending on the lipid composition, initial pressure, and amount of injected protein) upon injection of aSyn at constant area, followed by a large and steady increase in surface pressure (Figure 3a), suggesting that aSyn is highly surface‐active and is inserted into the lipid monolayer [9]. This is distinct from the effect of synapsin, which is followed by a temporary drop in surface pressure but no significant pressure increase over long time [35]. Interaction with negatively charged monolayers leads to a compaction of the monolayer (deviating from the monolayer isotherm)—possibly due to nonspecific interactions between the protein and the lipids, e.g., electrostatic repulsion between the anionic DPPS and the C‐terminal segment of aSyn. Despite the changes in the area per lipid (GID), no detectable changes were observed in the vertical density profile ρ(z) (XRR) for the DPPC/DPPS, and the DOPC/DOPS monolayers.

Contrarily, in the GM1‐containing film, aSyn injection led to detectable changes in ρ(z) (increase in head group density and decrease in tail group density), which we tentatively attribute to strong specific interactions between aSyn and GM1 [18, 20]. Note, however, that such a specific interaction cannot be directly inferred from the data but rather by interpretation. Future work including controls varying the GM1 mol fraction, headgroup‐selective mutants, or the well‐established GM1‐binding fragment (aa 34–50) discussed in the Introduction, as well as controls with aSyn lacking the ganglioside‐binding domain, could provide further evidence and possibly a mechanistic explanation. To avoid unwarranted generalization or conclusions drawn from the Langmuir experiments, we also want to include a warning: the absence of a substantial XRR response for the DPPC/DPPS monolayer does not yet imply that aSyn generally has only minor effects on fluid biological membranes in general. The DOPC/DOPS monolayer is more relevant in terms of chain fluidity, but the rather high surface pressure of 35 mN/m applied to obtain sufficient diffraction signal may also have prevented protein insertion. The results demonstrate surface activity and subtle changes in lateral lipid packing, but they do not provide a broadly applicable test of membrane disruption or remodeling. Of course, a constrained planar monolayer cannot report bending, budding, tubulation, or other shape transformations of a bilayer. On the other hand, a disruption of the lipid film could be clearly ruled out for the conditions tested.

In LVs with a *bona fide* lipid composition representative for SVs, the vesicle shape and bilayer structure are preserved in the presence of aSyn at physiological protein concentration. At low micromolar concentrations, Syn1 has a stronger effect on vesicle SAXS curves than aSyn, due to (i) the Syn1 induced clustering of vesicles (low‐q region) and (ii) an increased outer head group density (based on fits to the high‐q region). Rather than a direct consequence of protein electron density, this can possibly be explained by flattened adhesion zones of LVs creating lateral tension [34]. The fact that the vertical density profile of Langmuir lipid monolayers did not show any observable changes for DPPC:DPPS and DOPC:DOPS but only for the GM1 system can be contrasted with the bulk SAXS results, where the anionic lipid bilayer profile did change also in a GM1‐free model system. This difference is consistent with the reported curvature‐dependent binding and insertion behavior of aSyn. However, the here presented monolayer and vesicle experiments also differ in lipid composition, membrane geometry, and protein concentration. Finally, for SV condensates induced by Syn1 but in the presence of aSyn, we also observe a structure factor modulation indicative of short‐range order between SVs, as we reported before for Syn1 only [33]. Qualitative inspection of this preliminary data and comparison with the previous aSyn‐free SAXS lineshapes [33] point to a a noncompact liquid structure also in the presence of aSyn.

Future work could target several interesting questions raised by the current findings: In the monolayer protein interaction, what is the role of liquid‐expanded (LE) and liquid–crystalline (LC) domains? Does aSyn preferentially insert into the LE domains? If so, one can speculate that the LC phase, which dominates the GID signal, is compressed. To investigate whether the LC domains are growing or shrinking due to aSyn, one could, for example analyze the lineshapes of the GID peaks and determine the domain size. Note that for tau protein probed at 1 μM concentration and at constant area of the Langmuir film, LC domains were found to dissolve [55, 56]. The thermal phase behavior of the monolayer and the main phase transition of the lipid could also be shifted by interaction with aSyn. Concerning both model systems, planar membrane and LVs: an extension to higher aSyn concentration in the pathological range would be most interesting. To this end, a smaller volume than the trough would be imperative since the required protein amounts would otherwise be excessive. A small chamber with a single solid‐supported bilayer seems to be the most feasible approach for XRR and GID. For the vesicle‐protein system, it remains to be seen where the transition from the present structure‐preserving scenario to the disruptive behavior and break‐up into small, mostly disc‐ or rod‐like lipid‐protein particles reported by [49] takes place. Note that in the latter work, only pathologically high concentrations (70–270 μM) of aSyn were probed in contact with vesicles of very high negative surface charge density (100 mol% DLPS or DMPS). In terms of lipid composition, in our view, the PC/PS/GM1 system is particularly interesting for further study over a wide range of aSyn concentrations and also well‐suited to probe and compare different aSyn variants.

## 4 Experimental Methods

### 4.1 Sample Preparation

#### 4.1.1 Biochemicals

Lipids were purchased as powders from Avanti Research (Alabaster, AL, USA), including 1,2‐dipalmitoyl‐sn‐glycero‐3‐phosphocholine (DPPC, Avanti code: 850355P), 1,2‐dipalmitoyl‐sn‐glycero‐3‐phospho‐L‐serine (DPPS, Avanti code: 840037P), Ganglioside GM1 (Avanti code: 860065P), 1,2‐dioleoyl‐sn‐glycero‐3‐phosphocholine (DOPC, Avanti code: 850375P), 1,2‐dioleoyl‐sn‐glycero‐3‐phospho‐L‐serine (DOPS, Avanti code: 840035P), 1,2‐dioleoyl‐sn‐glycero‐3‐phosphoethanolamine (DOPE, Avanti code: 850725P), and cholesterol (Avanti code: 700000P). Fluorescently labeled lipids Marina Blue 1,2‐dihexadecanoyl‐sn‐glycero‐3‐phosphoethanolamine (Marina Blue DHPE) and Texas Red DHPE were purchased from Thermo Fisher Scientific (Waltham, MA, USA).

Untagged human aSyn was expressed in BL21(DE3) Escherichia coli cells and purified as previously described [37, 57]. The concentration of purified aSyn was determined by measuring the absorbance at 280 nm, employing an extinction coefficient of 5960 M^−1^cm^−1^ for aSyn. EGFP‐tagged human synapsin 1a (Syn1) was expressed in Expi293 cells (Thermo Fisher Scientific, MA, USA) and purified as previously described [37]. Proteins were snap‐frozen in liquid nitrogen and stored at −80 °C until further use. Proteins were always handled on ice to reduce protein activity before using it in a sample.

SVs were isolated from rat brains and purified as previously described [37]. After extraction, SVs were snap‐frozen and kept at −80 °C or in liquid nitrogen and were thawed on ice before the SAXS experiments.

TBS pH 7.4, composed of 25 mM Tris–HCl, 150 mM NaCl, and 0.5 mM TCEP, was used as the buffer for vesicles and proteins, as well as in the subphase below the lipid monolayers.

#### 4.1.2 LVs

LVs mimicking the size, phase state, and charge distribution of SVs were prepared from 55 mol% DOPC, 20 mol% DOPS, 15 mol% DOPE, and 10 mol% cholesterol by 10 freeze–thaw cycles and extrusion through a polycarbonate membrane of 50 nm pore size using an Avanti Mini Extruder (Avanti Research, AL, USA), as previously described [25, 34]. For fluorescence microscopy, 0.04 mol% Marina Blue DHPE was added. All lipid vesicle concentrations are given as pre‐extrusion lipid concentrations.

### 4.2 Synchrotron Experiments

#### 4.2.1 Langmuir Monolayer Setup

Langmuir experiments were conducted in a custom‐made Teflon Langmuir trough (10 × 13 × 0.42 cm^3^, $V_{\mathrm{subphase}}\;\approx \;55\;\mathrm{mL}$) equipped with a movable Teflon barrier for surface pressure control. Control X‐ray measurements on bare lipid monolayers without proteins were performed using a larger beamline‐specific Langmuir trough (17 × 41 ×0.6 cm^3^, $V_{\mathrm{subphase}}\;\approx 400\;\mathrm{mL}$). The surface pressure was measured with a calibrated Wilhelmy balance. Before every new filling, the trough was thoroughly cleaned with isopropanol and ultrapure water. To remove any remaining impurities, the trough was filled with ultrapure water, the Teflon barrier was closed, and the water surface was sucked off with a vacuum pump. This process was repeated until no rise in surface pressure was observable during compression of a bare surface (ΔΠ<0.2 mN/m). To prepare lipid monolayers, lipid stock solutions (prepared in a 2:1 mix of chloroform and methanol at a lipid concentration of 0.2 mg/mL in the desired molar ratios) were carefully spread on the buffer surface with a microsyringe (Hamilton, NV, USA) and allowed to equilibrate for at least 15 min while monitoring the surface pressure. After experiments involving aSyn in the subphase, the Langmuir trough was first cleaned with 1% (w/v) sodium dodecyl sulfate (SDS) solution as a safety precaution to inactivate any potentially misfolded aSyn aggregates [58, 59], followed by the standard cleaning steps with isopropanol and ultrapure water.

X‐ray experiments on the Langmuir monolayer were carried out at the ID10‐SURF beamline of the European Synchrotron Radiation Facility (ESRF) in Grenoble [60, 61]. The ESRF was operating in 7/8 + 1 filling mode, and the three ID10 undulators were tuned in series to generate a synchrotron beam of high brilliance, which was subsequently monochromatized to 22.5 keV by a Si(111) channel‐cut monochromator. The beam was focused by a set of 32 Be compound refracting lenses (CRLs) to $27\;\times \;40\;{\mathrm{\mu m}}^{2}$ (vertical × horizontal) beam size with a flux of approximately $10^{12}$ photons/s and tilted by a double‐crystal deflector (DCD) before impinging on the sample. The Langmuir trough was placed on an antivibration stage and kept under water‐saturated helium to reduce parasitic scattering. All measurements were done at a set temperature of 23 °C. The trough was translated by 0.5 mm after each measurement set (XRR + GID), and the resulting curves were checked for radiation damage.

GID scans were recorded at an angle of incidence of 0.044° (80% of the critical angle) using a Mythen2K linear photon‐counting detector positioned approximately 560 mm behind the sample. A Soller collimating slit with a resolution of 0.08° was placed before the detector to reduce the noise from parasitic scattering. The exposure time was 5 s at each value of the lateral momentum transfer $q_{xy}$.

XRR scans were recorded using a 2D CdTe Maxipix detector equipped with an evacuated flight tube with two sets of collimating slits at the entry and exit points to select in‐plane specularly reflected photons. To improve the XRR signal‐to‐noise ratio at high $q_{z}$, the full $q_{z}$‐range of 0–0.8 Å^−1^ was split into six consecutive scans with increasing exposure time towards higher angles (from 1 s per scan point at low $q_{z}$ up to 30 s per scan point between $q_{z}≃0.6$ to 0.8 Å^−1^), which were later converted into a single dataset by stitching the adjacent (overlapping) $q_{z}$‐regions after proper normalization. The beamtime data (LS‐3508) is publicly available under https://doi.esrf.fr/10.15151/ESRF‐ES‐2159193414.

#### 4.2.2 SAXS Experiments

SAXS experiments were carried out at the BM26 beamline (DUBBLE) of the ESRF in Grenoble. The ESRF was operating in 7/8 + 1 filling mode, and a synchrotron beam of high brilliance was generated by the bending magnet of BM26, which was collimated and subsequently monochromatized to 13 keV by a double‐crystal monochromator with sagittal focusing. The beam was further focused vertically to a beam size of about $350\;\times \;700\;{\mathrm{\mu m}}^{2}$ (vertical× horizontal) with a flux of approximately $10^{10}$ photons/s at the sample. SAXS data were recorded at full beam intensity using a Pilatus 1 M single‐photon counting pixel detector (DECTRIS, Baden‐Dättwil, Switzerland). The primary beam was blocked by a beamstop. To reduce air scattering, an evacuated flight tube was placed between the sample holder and the detector. A silver behenate (AgBeh) calibration standard was used to calibrate the sample‐to‐detector distance (∼3m) and the detector q‐range, where q is defined as $q=\frac{4\pi }{\lambda }\sin\theta$. The samples were mixed on ice in Eppendorf PCR tubes, and between 10 and 30 μL sample solution was filled into quartz capillaries of ∼2 mm diameter and ∼0.01 mm wall thickness (Hilgenberg GmbH, Malsfeld, Germany). The capillaries were placed horizontally in the air gap between the scatterless guard slits and the flight tube. The accumulation time was set to either 30 or 60 s per sample. A buffer capillary containing only TBS was mounted on each sample holder, and the SAXS pattern of the pure buffer was measured before the corresponding sample. During the measurements, the 2D scattering patterns were simultaneously normalized and azimuthally averaged by the online data reduction tool BUBBLE [62]. The beamtime data (LS‐3508) is publicly available under https://doi.esrf.fr/10.15151/ESRF‐ES‐2159193457. Additional data (Figure 7) for the revision of the MS was recorded at BM29 [54] in beamtime MX‐2851 (https://doi.esrf.fr/10.15151/ESRF‐ES‐2457160090).

### 4.3 X‐Ray Data Analysis

#### 4.3.1 GID

Each separately recorded 1D intensity profile $I_{q_{xy}}(q_{z})$ was combined into a 2D intensity map $I(q_{z},q_{xy})$, covering the in‐plane and perpendicular components of the scattering vector $q_{z}=\frac{4\pi }{\lambda }\sin\alpha_{f}$ and $q_{xy}=\frac{4\pi }{\lambda }\sin\theta$, respectively. For monolayers containing saturated lipids (Figure 3–4), two first‐order peaks are observed at two distinct values of $q_{xy}$, suggesting a distorted hexagonal chain lattice that can be indexed in terms of a centered rectangular unit cell [63]. The positions of the chain lattice peaks were determined by least‐square fitting, as detailed in the Supporting Information. The $q_{xy}$‐components of these peaks correspond to 2D lattice spacings $d_{hk}=2\pi /q_{xy}^{hk}$, indexed by the Miller indices h and k. The lattice spacings in turn determine the unit cell parameters (a, b) and the area per lipid chain $A_{c}$ [64].

(2) $$\begin{array}{c} A_{c}\;=\;\frac{ab}{2}=\frac{d_{02}}{\sqrt{{\left(\frac{1}{d_{11}}\right)}^{2}-{\left(\frac{1}{2d_{02}}\right)}^{2}}}\; \end{array}$$

The vertical components ($q_{z}^{11}>0$ and $q_{z}^{02}\approx 0$ Å^−1^) suggest that the chains tilt towards one of their nearest neighbors (NN‐tilted phase) [63]. The tilt angle τ can be calculated given the value of $q_{z}^{11}$ and is shown only in the Supporting Information, because it is highly correlated with $A_{c}$.

#### 4.3.2 XRR

Raw XRR data were processed using in‐house developed scripts at the ID10‐SURF beamline to adjust for exposure time, illumination correction, attenuators, and background. After background subtraction and proper normalization, the six consecutive scans covering the full $q_{z}$‐range of 0 to 0.8 Å^−1^ were converted into a single dataset by stitching together the adjacent (overlapping) $q_{z}$‐regions. The stitched dataset was normalized by the direct beam intensity $I_{0}$ (measured in a separate scan immediately before the corresponding XRR scan) to derive the intensity reflectivity $R(q_{z})\;=\;I_{R}(q_{z})/I_{0}$. Data points where the background noise exceeded the signal intensity were excluded from further analysis. XRR fitting was performed using the differential evolution algorithm implemented in GenX [65]. The “logR2” figure of merit (FOM), a logarithmic modification of the crystallographic R2 factor, was used for fitting, which uses log‐space residuals to enable good fits across the entire $q_{z}$‐range covering up to 11 orders of magnitude in intensity $R(q_{z})$. The density profiles of the buffer‐lipid‐air interface ρ(z) were modeled as a layered structure of 2 or 3 layers on top of a semi‐infinite subphase, and the specular reflectivity was simulated using the Parratt recursive formalism [66]. The 2 upper layers represent the lipid tail groups and head groups, while for GM1‐containing monolayers, a third layer was introduced representing the glycan portion of the GM1 head group. For each layer, the layer density ρ and length L were allowed to vary freely. In addition, the interfacial roughness was modeled via the Nevot–Croce factor [67], which results in an exponential damping of reflectivity at each interface i, $\propto \exp(\text{−}\frac{q_{z}^{2}\sigma_{i}^{2}}{2})$, with root mean square roughness $\sigma_{i}$ that was also varied during fitting. In total, the 2‐layer model had 7 free parameters (2 layers and 3 interfaces) and the 3‐layer model had 10 free parameters (3 layers and 4 interfaces).

#### 4.3.3 SAXS

The 2D SAXS patterns were azimuthally averaged and normalized using pyFAI [68]. Repeated measurements of the same sample at different positions along the capillary were averaged and the background SAXS curve of the buffer capillary was subtracted to yield the scattering functions I(q) plotted in Figures 6a and 8a,c. The fitting of the SAXS curves is detailed in the Supporting Information.

## Funding

This study was supported by Deutsche Forschungsgemeinschaft (CRC 1286 A02, B08, and B10; Project number 317475864 and Project‐ID 449750155) and German Federal Ministry of Research, Technology, and Space (BMFTR) (MPS‐Matter to Life).

## Conflicts of Interest

The authors declare no conflicts of interest.

## Supporting information

Supplementary Material

## Data Availability

The data that support the findings of this study are openly available in ESRF Data portal at https://doi.esrf.fr/10.15151/ESRF‐ES‐2159193414, reference number LS‐3508.
