# Supplementary material for: Phospholipid Monolayers and Vesicular Membranes Show Only Minor Structural Changes After Interaction With α‐Synuclein at Low and Physiological Concentrations
Source: Chemphyschem. 2026 Oct 22;27(20):e70589. doi: 10.1002/cphc.70589 (PMC13691286; doi:10.1002/cphc.70589)
Supplement: Supplementary file 1 [file CPHC-27-e70589-s001.pdf]

Supporting Information for

Phospholipid monolayers and vesicular membranes show only minor structural changes after interaction with α-synuclein at low and physiological concentrations

András Major[a], Eva von Campenhausen[a], Riko Hilger[a], Hendrik Bruns[a], Titus Czajka[a], Diego Pontoni[b], Martin Rosenthal[b], Gerard Aguilar Pérez[c], Christian Hoffmann[c], Mohammed Al-Azzani[d], Tiago F. Outeiro[d,e], Dragomir Milovanovic[c], Tim Salditt*[a]

Experimental Methods

Grazing-incidence diffraction (GID) peak fitting and lattice parameters

The GID data presented in the main MS is the result of fitting the {11} and {02} lipid chain lattice peaks in the (q_{xy}, q_z)-plane, as described previously [1]. The lateral ordering of the lipid chains in liquid-condensed domains can be described as a two-dimensional (2D) crystal in terms of a centered

[a] A. Major, E. v. Campenhausen, R. Hilger, H. Bruns, T. Czajka, Prof. Dr. T. Salditt*
Institute for X-Ray Physics
University of Göttingen
Friedrich-Hund-Platz 1, 37077 Göttingen, Germany
E-mail: tsaldit@gwdg.de

[b] Dr. D. Pontoni
ESRF - The European Synchrotron
71 avenue des Martyrs, 38000 Grenoble, France

[c] Dr. M. Rosenthal
Department of Chemistry
KU Leuven
Celestijnenlaan 200f, 3001 Leuven, Belgium

[d] Dr. G. A. Pérez, Dr. C. Hoffmann, Prof. Dr. D. Milovanovic
Laboratory of Molecular Neuroscience, DZNE - German Center for Neurodegenerative Diseases,
Institute of Biochemistry, Charité - Universitätsmedizin Berlin, Corporate Member of Freie Universität Berlin, Humboldt-Universität Berlin, and Berlin Institute of Health
Virchowweg 6, 10117 Berlin, Germany

[e] Dr. M. Al-Azzani, Prof. Dr. T. F. Outeiro
Department of Experimental Neurodegeneration
Center for Biostructural Imaging of Neurodegeneration
University Medical Center Göttingen
Waldweg 33, 37073 Göttingen, Germany

[e] Prof. Dr. T. F. Outeiro
Faculdade de Medicina e Ciências Biomédicas
Algarve Biomedical Center Research Institute (ABC-Ri)
Universidade do Algarve
8005-139 Faro, Portugal

Translational and Clinical Research Institute
Faculty of Medical Sciences
Newcastle University
Framlington Place, Newcastle Upon Tyne, NE2 4HH, UK

Scientific employee with an honorary contract at Deutsches Zentrum für Neurodegenerative Erkrankungen (DZNE)
Göttingen, Germany

rectangular unit cell [2], with lattice vectors a and b. The lattice spacings d_{hk}, indexed by the Miller indices h and k, are related to the in-plane q_{xy}-components of the the diffraction peaks $d_{hk} = 2\pi/q_{xy}^{hk}$. From the Laue equations it follows that the lattice spacings of a rectangular lattice are related to the lattice vectors as [3]

$$\frac{1}{d_{hk}} = \frac{\sqrt{h^2b^2 + k^2a^2}}{ab}\,, \tag{1}$$

where $(h + k)$ is an even integer for a centered rectangular lattice [3]. From $d_{02} = 2\pi/q_{xy}^{02}$, we can directly infer the lattice parameter b:

$$\frac{1}{d_{02}} = \frac{\sqrt{0^2b^2 + 2^2a^2}}{ab} = \frac{2}{b} \quad \Rightarrow \quad b = 2d_{02}\,. \tag{2}$$

This relation for b can then be inserted in the expression for d_{11} in Eq.(1)

$$\frac{1}{d_{11}} = \frac{\sqrt{1^2b^2 + 1^2a^2}}{ab} = \sqrt{\frac{1}{a^2} + \frac{1}{b^2}} \overset{(2)}{=} \sqrt{\frac{1}{a^2} + \left(\frac{1}{2d_{02}}\right)^2} \tag{3}$$

$$\Rightarrow \quad \frac{1}{a^2} = \left(\frac{1}{d_{11}}\right)^2 - \left(\frac{1}{2d_{02}}\right)^2 \tag{4}$$

$$\Rightarrow \quad a = \frac{1}{\sqrt{\left(\frac{1}{d_{11}}\right)^2 - \left(\frac{1}{2d_{02}}\right)^2}}\,. \tag{5}$$

From the lattice vectors a and b, one obtains the area per chain $A_c = \frac{ab}{2}$, presented in the main MS. Given the out-of-plain component of the $\{11\}$ peak, q_z^{11}, the tilt angle τ with respect to the surface normal can be further calculated [2]

$$\tan(\tau) = \frac{q_z^{11}}{\sqrt{(q_{xy}^{11})^2 - \left(\frac{q_{xy}^{02}}{2}\right)^2}}\,. \tag{6}$$

The corresponding 2D-measurements in the (q_{xy}, q_z)-plane are shown in Fig. S5, S6, S7. The dark lines indicate the fit results for the three relevant peak positions (q_z^{11}, q_{xy}^{11}, q_{xy}^{02}). The peak center is determined by projecting the intensity onto the respective axis and subsequently fitting a Lorentzian $\frac{c\cdot\sigma^2}{(q-\hat{q})^2+\sigma^2}$ to the data, where q is the independent variable (either q_{xy} or q_z), c is a scaling factor related to the peak height, $\hat{q}$ represents the peak center position used to calculate the lattice spacings, and the width σ is used to determine the error.

SAXS models and data analysis

Flat membrane model

Here, it is assumed that the signal is dominated mainly by the EDP, so a corresponding microscopic picture would show randomly orientated flat membrane snippets floating in the buffer. An equivalent scattering curve is found e.g. for cell-sized giant unilamellar vesicles. Based on [4], the model function is in this case

$$F_{\text{flat}}(q) = \frac{1}{q^2}\sum_{i=1}^{3}\sum_{j=1}^{3}\rho_i\rho_j\sigma_i\sigma_j \exp\Big[-\frac{q^2(\sigma_i^2+\sigma_j^2)}{2}\Big]\cos[q(z_i-z_j)]. \tag{7}$$

Polydisperse spherical vesicle model

The vesicles are modeled as polydisperse perfect spheres with radius R and a Gaussian polydispersity function with a width σ_R. Relying on [4], we have an approximate model function without numerical computation at our disposal:

$$F_{\text{sphere}}(q) = \frac{1}{q^2}\sum_{i=1}^{3}\sum_{j=1}^{3}\rho_i\rho_j\sigma_i\sigma_j \exp\Big[-\frac{q^2(\sigma_i^2+\sigma_j^2)}{2}\Big][A_{ij}(q)-B_{ij}(q)+C_{ij}(q)] \tag{8}$$

with

$$A_{ij}(q) = [(R+z_i)(R+z_j)+\sigma_R^2]\cos[q(z_i-z_j)], \tag{9}$$
$$B_{ij}(q) = [(R+z_i)(R+z_j)+\sigma_R^2-4q^2\sigma_R^4]\cos[q(2R+z_i+z_j)]\exp(-2q^2\sigma_R^2), \tag{10}$$
$$C_{ij}(q) = 2q\sigma_R^2\exp(-2q^2\sigma_R^2)(2R+z_i+z_j)\sin[q(2R+z_i+z_j)]. \tag{11}$$

Polydisperse ellipsoidal vesicle model

This model introduces an ellipticity ε and a corresponding Gaussian polydispersity with width σ_ε to account for relevant deviations from the spherical shape. More specific, the vesicles are imagined as rotational ellipsoids or spheroids with two equal axes R and one axis $\varepsilon \cdot R$. By defining a variable $u(R) = q \cdot R \cdot \sqrt{\sin^2(\vartheta)+\varepsilon^2\cos^2(\vartheta)}$, one can derive the model function analytically up to the polydispersity integrals directly from the canonical form factor of a homogeneous sphere (see [5]). The result of this lengthy calculation is

$$F_{\text{ellipsoid}}(q) = \int_{\varepsilon-3\sigma_\varepsilon}^{\varepsilon+3\sigma_\varepsilon}\frac{\mathrm{e}^{-\frac{(\varepsilon'-\varepsilon)^2}{2\sigma_\varepsilon^2}}}{\sigma_\varepsilon\sqrt{2\pi}}\int_{R-3\sigma_R}^{R+3\sigma_R}\frac{\mathrm{e}^{-\frac{(r-R)^2}{2\sigma_R^2}}}{\sigma_R\sqrt{2\pi}}\int_0^{\pi/2}|f(q,r,\varepsilon',\vartheta)|^2\sin(\vartheta)\,\mathrm{d}\vartheta\,\mathrm{d}r\,\mathrm{d}\varepsilon' \tag{12}$$

with

$$\begin{aligned} f(q,R,\varepsilon,\vartheta) =&\varepsilon\Big[\rho_1\sigma_1\exp\Big(-\frac{\sigma_1^2u^2(R)}{2R^2}\Big)\Big(\frac{(R-z_h)^2\sin(u(R-z_h))}{u(R-z_h)}+\sigma_1^2\cos(u(R-z_h))\Big)\\ &+\rho_c\sigma_c\exp\Big(-\frac{\sigma_c^2u^2(R)}{2R^2}\Big)\Big(\frac{R^2\sin(u(R))}{u(R)}+\sigma_c^2\cos(u(R))\Big)\\ &+\rho_3\sigma_3\exp\Big(-\frac{\sigma_3^2u^2(R)}{2R^2}\Big)\Big(\frac{(R+z_h)^2\sin(u(R+z_h))}{u(R+z_h)}+\sigma_3^2\cos(u(R+z_h))\Big)\Big]. \end{aligned} \tag{13}$$

The limits of the polydispersity integrals should formally go from 0 to ∞, but the 3σ environment is a sufficient approximation.

Fluorescence microscopy of vesicle-protein condensates

Fluorescence microscopy experiments (Fig.2 of the main manuscript) were carried out using a Zeiss Axio Observer Z1 inverted microscope (Zeiss, Jena, Germany). Chemical labeling of aSyn for fluorescence microscopy was performed with a 10-times molar excess of the NHS-ester reactive fluorescent Alexa Fluor 647 dye (AF647, Thermo Fisher Scientific, MA, USA) following the protocol described in [6]. LVs, aSyn and/or Syn1 proteins were mixed on ice in the desired concentrations and pipetted into self-constructed sample chambers consisting of two glass coverslips connected by parafilm. Marina Blue DHPE was excited at $\lambda = 365\,\text{nm}$, EGFP-tagged Syn1 was excited at $\lambda = 470\,\text{nm}$, aSyn-AF647 was excited at $\lambda = 640\,\text{nm}$, and images were acquired with a monochromatic Axiocam MRm CCD camera (Zeiss, Jena, Germany). Spectral crosstalk can be excluded owing to the negligible spectral overlap of the three fluorophores and the use of non-overlapping bandpass filters.

In contrast to Syn1 [7], aSyn alone does not induce micron-scale condensate formation at low micromolar protein concentrations in the presence of 15 mM LVs, see Fig. S2, in line with previous experiments using purified SVs [6].

Supporting calculation: aSyn-membrane binding

Next we consider the fraction of membrane-bound protein f_b and the fraction of occupied 'effective' protein binding sites on the membrane f_o, as a function of bulk cytosolic concentration $[P]_{tot}$ of aSyn. For example, in the main MS we associated the concentration range $[P]_{\text{tot}} \leq 20\,\mu\text{M}$ with physiologically relevant bulk concentration values in the synapse, while we attributed $[P]_{\text{tot}} \geq 20\,\mu\text{M}$ to the pathological case. But in view of possible depletion effects, it is important to estimate the membrane-bound protein fraction

$$f_{\text{b}} = \frac{[PB]}{[P]_{\text{tot}}} \ , \tag{14}$$

and in view of the effect on membrane structure also the fraction of occupied membrane binding sites

$$f_{\text{o}} = \frac{[PB]}{[B]_{\text{tot}}} \ , \tag{15}$$

based on simple chemical reaction kinetics (Langmuir isotherm). Here, $[B]$ denotes the concentration of membrane binding sites, $[P]$tot the total protein concentration, as prepared in the trough or the vial, and $[PB]$ the unknown concentration of membrane-bound protein. Chemical equilibrium is given by the dissociation constant

$$K_D = \frac{[P][B]}{[PB]} \ , \tag{16}$$

and hence by combination of the expression above, we obtain

$$[PB] = \frac{[P]_{\text{tot}} + [B]_{\text{tot}} + K_D - \sqrt{([P]_{\text{tot}} + [B]_{\text{tot}} + K_D)^2 - 4[P]_{\text{tot}}[B]_{\text{tot}}}}{2}. \tag{17}$$

For K_D, we use the value determined by Maltseva et al. [8] in their quantitative αSyn binding assay. By titrating αSyn with increasing concentrations of lipid vesicles and fitting the resulting tryptophan-fluorescence, Maltseva et al. determined the membrane-bound fraction and analyzed their data with a lipid-depletion binding model. Importantly, Maltseva et al. used the same lipid composition as in our SAXS experiments (55 mol% DOPC, 20 mol% DOPS, 15 mol% DOPE, 10 mol% cholesterol), and vesicles prepared by extrusion through 50-nm pores. Following [8], we use the parameter σ, which specifies the number of effective membrane binding sites per lipid, such that

$$[B]_{\text{tot}} = \sigma[L]_{\text{tot}} \ , \tag{18}$$

where $[L]_{\text{tot}}$ denotes the total lipid concentration. From the protein-vesicle binding assay, Maltseva et al. obtained values of $\sigma = 0.0009\,\text{lipids}^{-1}$ and $K_d = 4.5\,\mu\text{M}$ (per lipid). For a complete membrane binding site, the dissociation constant has to be scaled (effective dissociation constant)

$$K_d \to \sigma K_d = 0.00405\,\mu\text{M} \ , \tag{19}$$

which is the value which we use in the binding-site equation above and the figure below. For the present SAXS experiments, the total lipid concentration was $[L]_{\text{tot}} = 15,\text{mM}$. Thus, the corresponding concentration of effective binding sites is $[B]_{tot} = 9.0 \times 10^{-4} \cdot 15\text{mM} = 13.5\mu\text{M}$. Fig. S1 shows the corresponding f_o, and f_b as a function of $[P]_{tot}$, plotted for the relevant concentration range of aSyn.

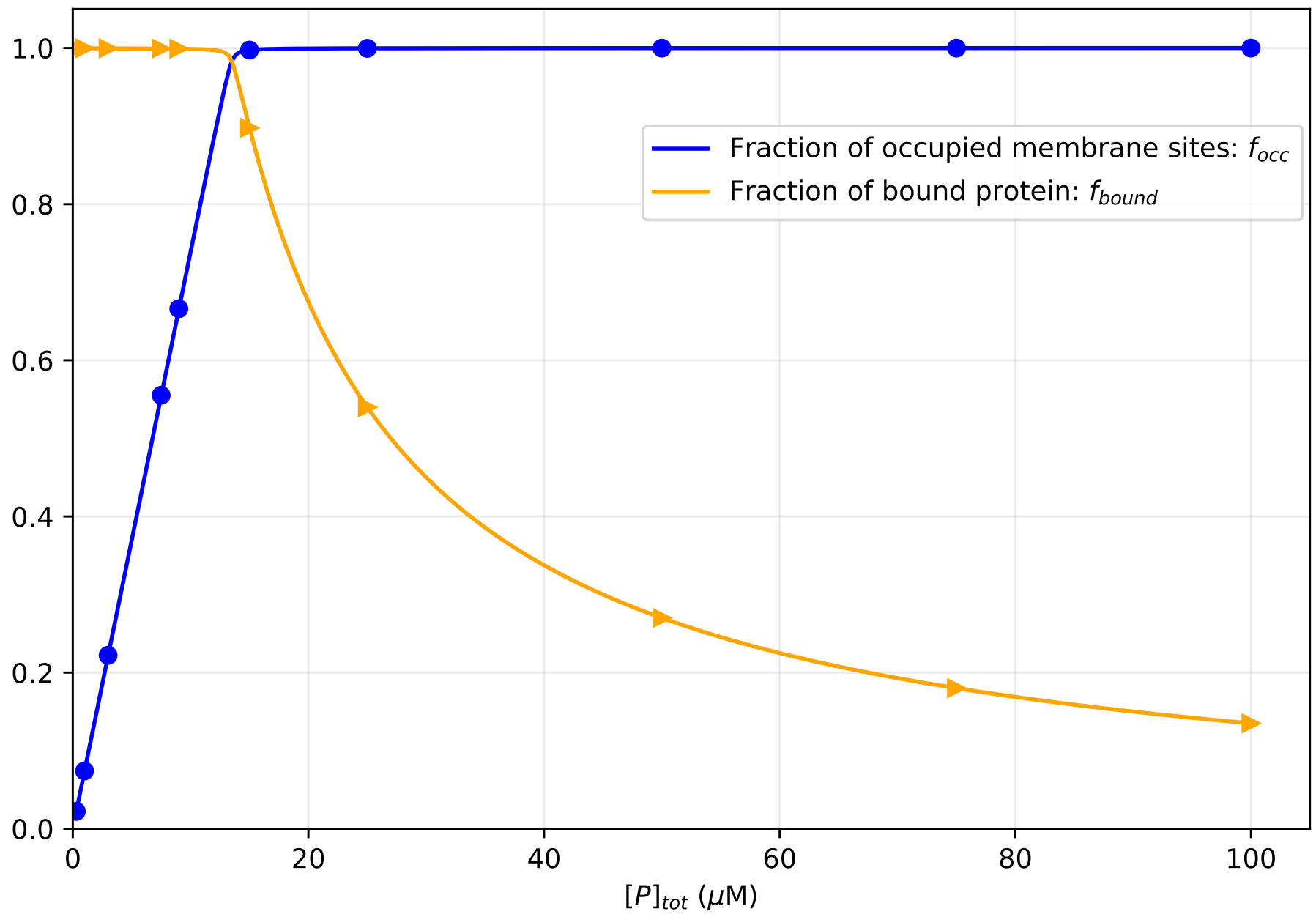

Fig. S1: The fraction of occupied membrane sites f_o (blue curve) and and the fraction of bound protein f_b (orange) as a function of protein concentration. The markers represent concentrations at which data was taken.

For the lipid monolayer experiments, we use the same binding model to obtain an order-of-magnitude estimate of the expected membrane-bound fraction. Since the binding-site density σ reported by Maltseva et al. is given per lipid bilayer, we approximated the corresponding binding-site density for a monolayer by multiplying σ by two, which gives $\sigma_{\text{monolayer}} = 1.8 \times 10^{-3}$ sites per lipid. For the Langmuir trough experiments we observed three different monolayers at two different αSyn concentrations. The total lipid concentration was calculated from the mass concentration of the injected vesicle solution, taking into account the molar mass corresponding to each lipid composition. The resulting number of moles divided by the trough volume $V = 55\text{ml}$ yields the total lipid concentration. The resulting fraction of occupied membrane and bound protein are summarized in table S1.

Lipids	$[L]_{\text{tot}}$ (μM)	$[P]_{\text{tot}}$ (nM)	f_{occ}	f_{bound}
DPPC:DPPS (1:1)	1.07	1	0.09	0.18
DPPC:DPPS (1:1)	1.07	10	0.53	0.10
DPPC:DPPS:GM1 (2:1:1)	0.849	10	0.53	0.08
DOPC:DOPS (1:1)	1.00	10	0.53	0.09

Table S1: Estimated αSyn membrane binding for the lipid monolayer experiments.

For the Langmuir trough, in particular, the resulting estimates should be interpreted with caution, since the K_D and σ values reported by Maltseva et al. were obtained under different experimental conditions with respect to membrane curvature, lipid composition and lateral surface pressure. Nevertheless, the estimate shows that already at 10nM αSyn we can expect a substantial fraction of membrane binding sites to be occupied.

Supporting Data

Fluorescence microscopy of LVs + aSyn

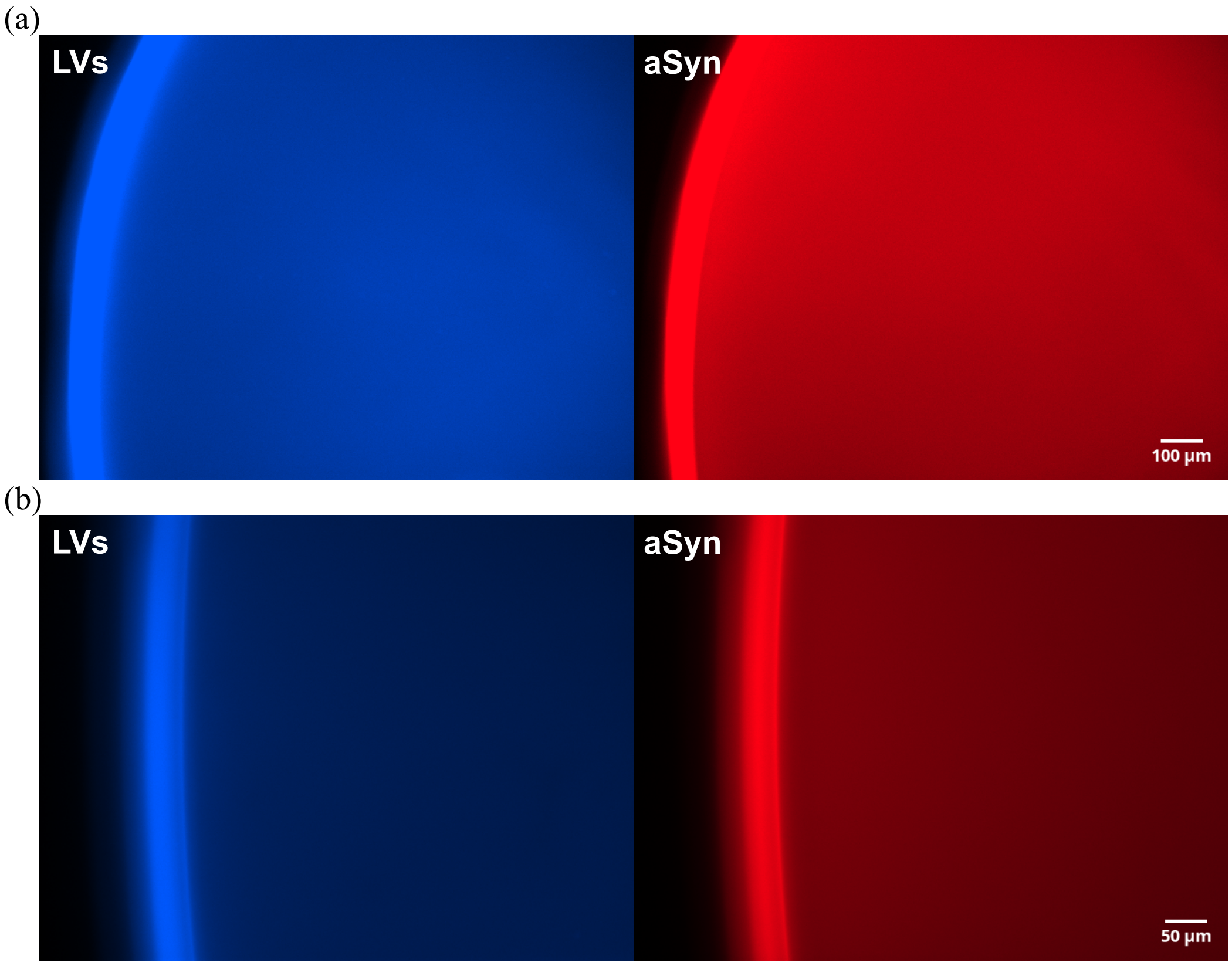

Fig. S2: aSyn does not induce vesicle condensation within the studied concentration regime. Both fluorescence channels show homogeneous intensity within the sample, the edge of the droplet is visible as an intense ring. (a) 15 mM LVs (labelled with 0.04 mol% Marina Blue DHPE) + 3 µM aSyn (equimolar amounts of unlabeled and Alexa Fluor 647-labeled). (b) 15 mM LVs + 9 µM aSyn (equimolar amounts of unlabeled and Alexa Fluor 647-labeled).

Instant pressure response to the injections

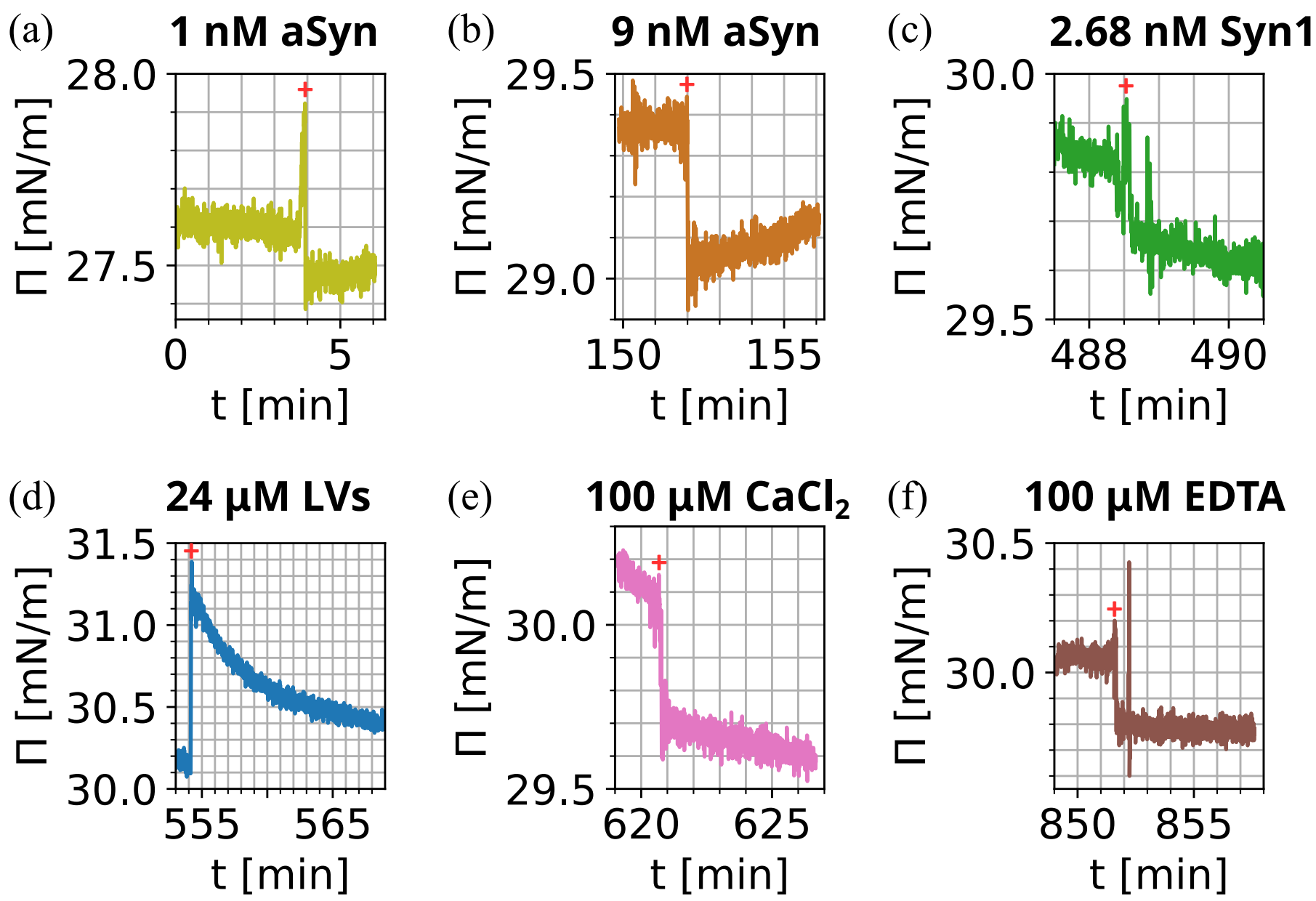

Fig. S3: Instant pressure response of the DPPC/DPPS (1:1) monolayer to the injections shown in Fig. 3.

GID data from the DOPC/DOPS monolayer

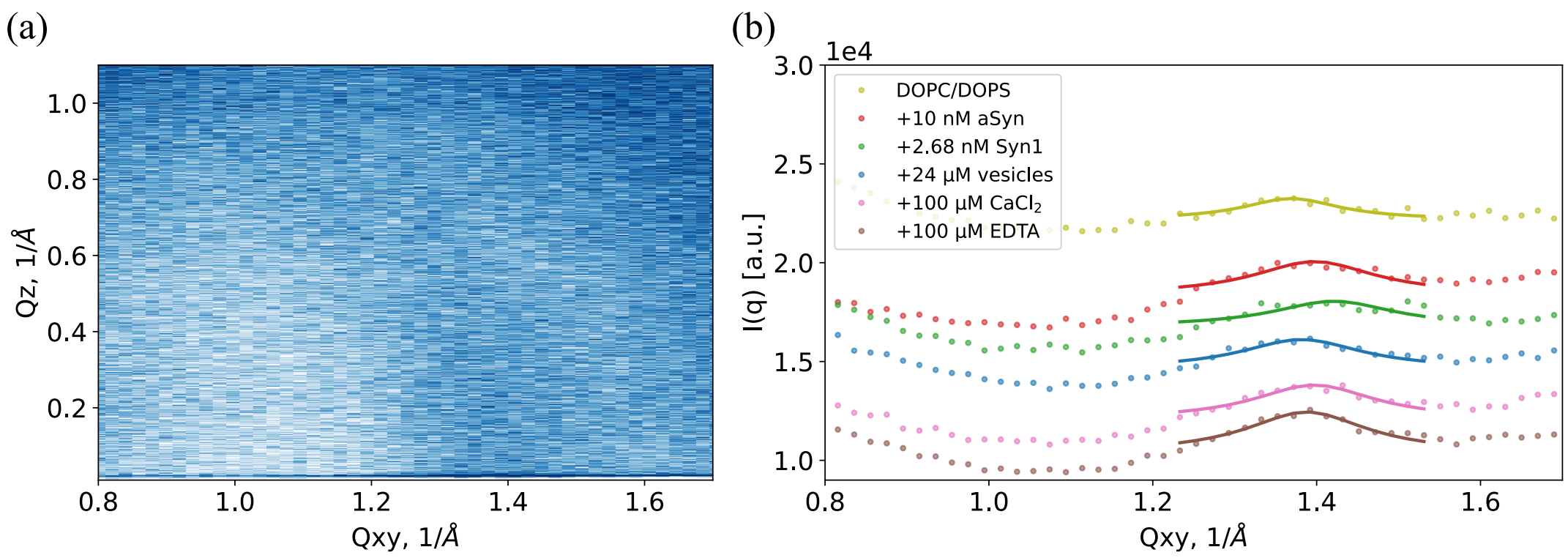

Fig. S4: GID data from the unsaturated DOPC/DOPS (1:1) monolayer, measured at constant surface pressure. (a) 2D GID data showing one fluid acyl chain correlation peak close to the $q_z = 0$ axis and $q_{xy} \simeq 1.4\,\text{Å}^{-1}$. (b) 1D GID data integrated over q_z for the same injection series as in Fig. 5.

GID fits

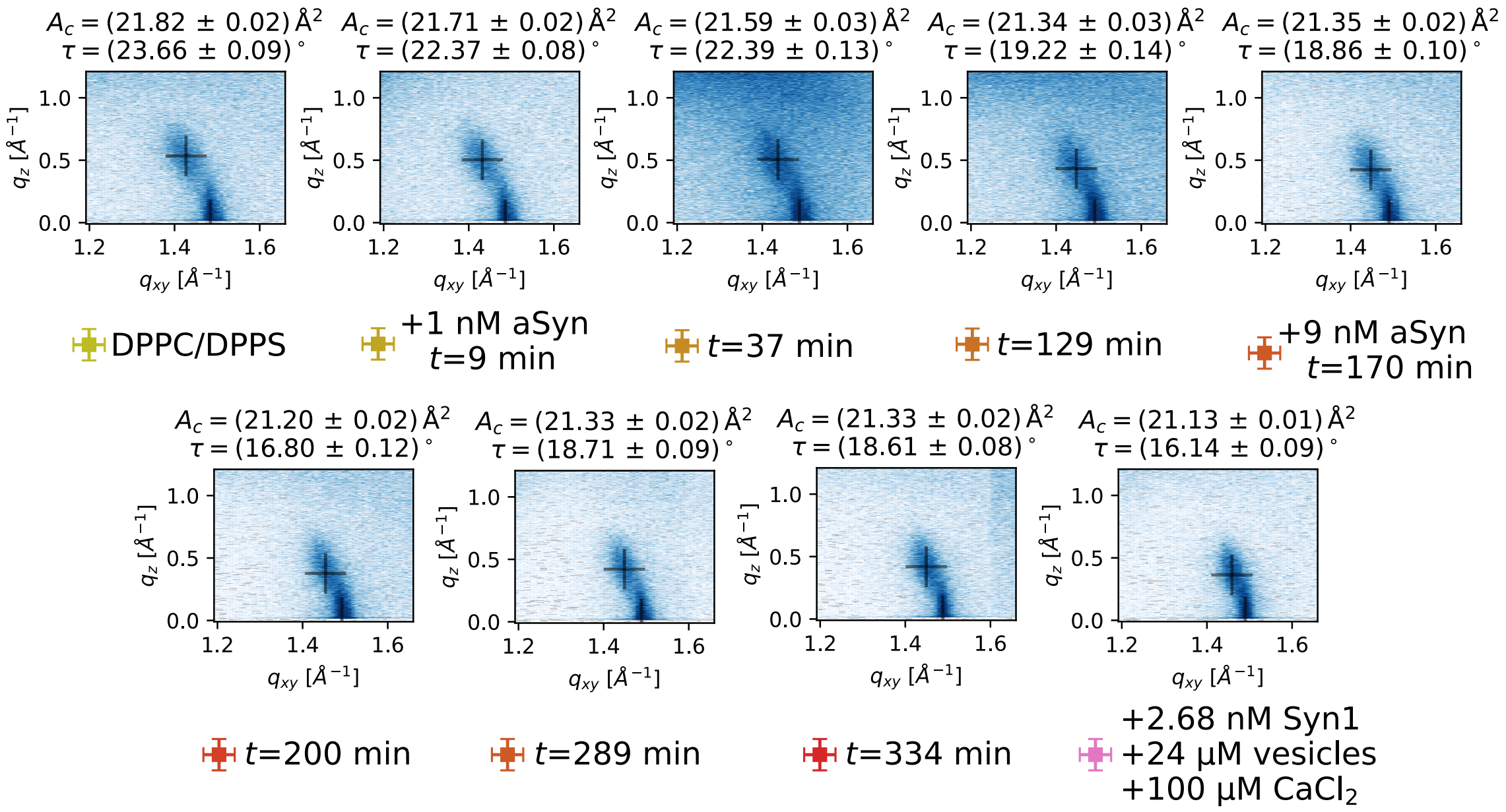

Fig. S5: GID data of DPPC/DPPS (1:1) corresponding to Figs. 3b-c. Two peaks are observed, the peak positions are indicated by the gray lines. The Lorentzian fits to the 1D integrated peak profiles are shown in Figs. S8 and S9. From the fitted peaks (q_z^{11}, q_{xy}^{11}, q_{xy}^{02}) the area per lipid chain A_c and the tilt angle τ are computed (see subpanel titles).

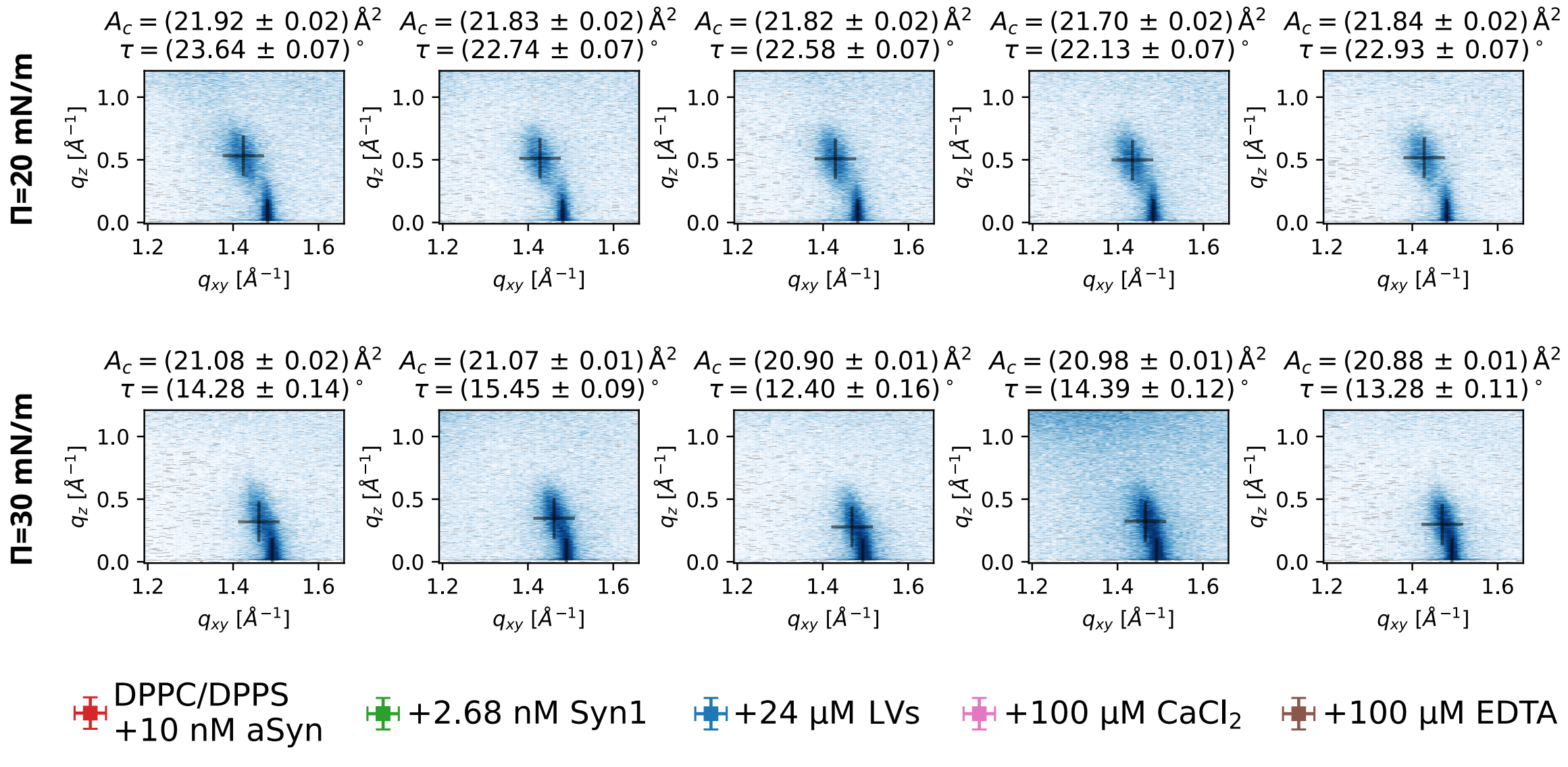

Fig. S6: GID data of DPPC/DPPS (1:1) corresponding to Fig. 3d. Upper row: GID data measured at $\Pi = 20\,\mathrm{mN/m}$ with peak positions (gray lines) corresponding to the fits in Fig. S10. Lower row: GID data measured at $\Pi = 30\,\mathrm{mN/m}$ with peak positions (gray lines) corresponding to the fits in Fig. S11. From the fitted peaks (q_z^{11}, q_{xy}^{11}, q_{xy}^{02}) the area per lipid chain A_c and the tilt angle τ are computed (see subpanel titles).

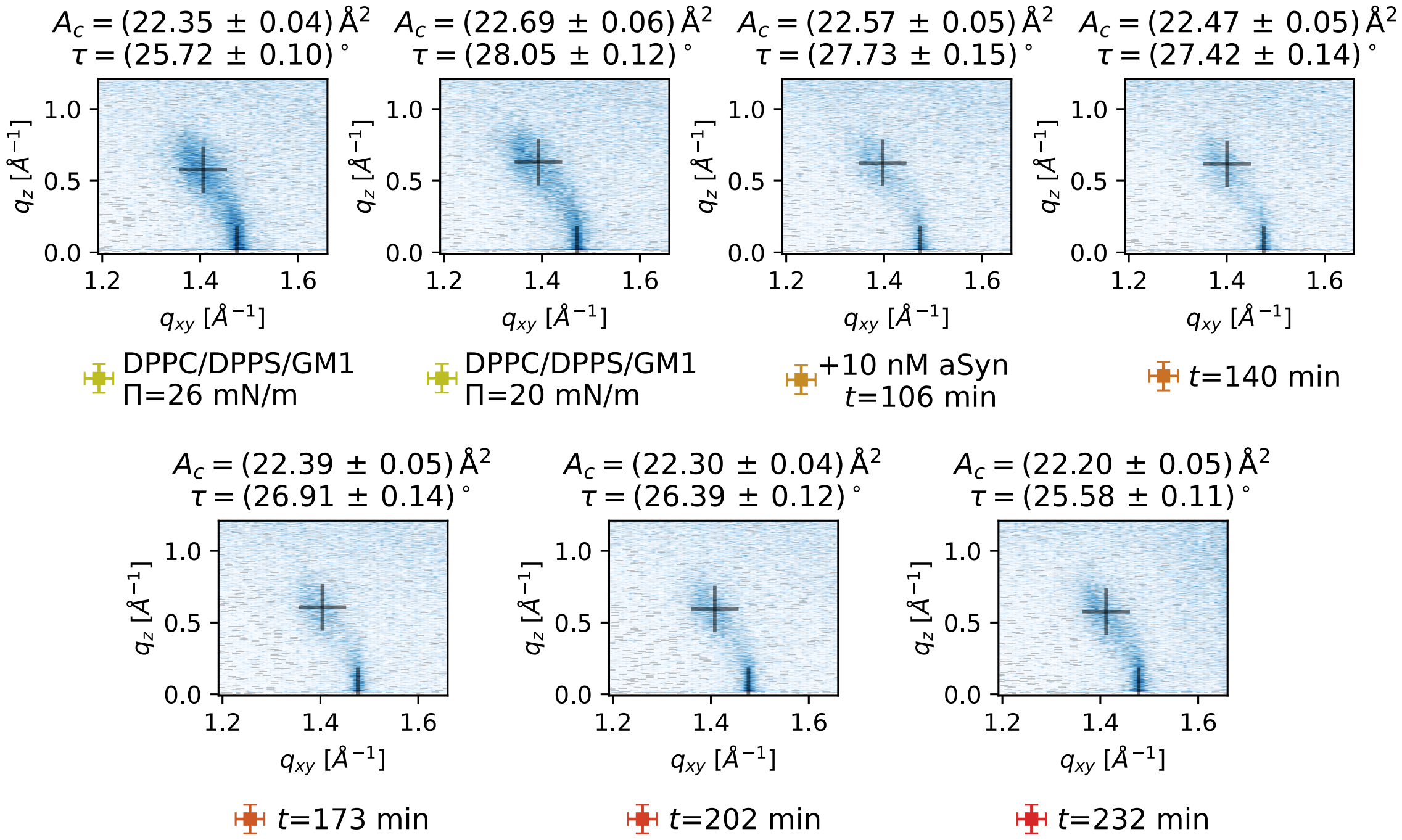

Fig. S7: GID data of DPPC/DPPS/GM1 (2:1:1) measured at constant monolayer area corresponding to Fig. 4b. Two peaks are observed, the peak positions are indicated by the grey lines. The Lorentzian fits to the 1D integrated peak profiles are shown in Fig. S12. From the fitted peaks (q_z^{11}, q_{xy}^{11}, q_{xy}^{02}) the area per lipid chain A_c and the tilt angle τ are computed (see subpanel titles).

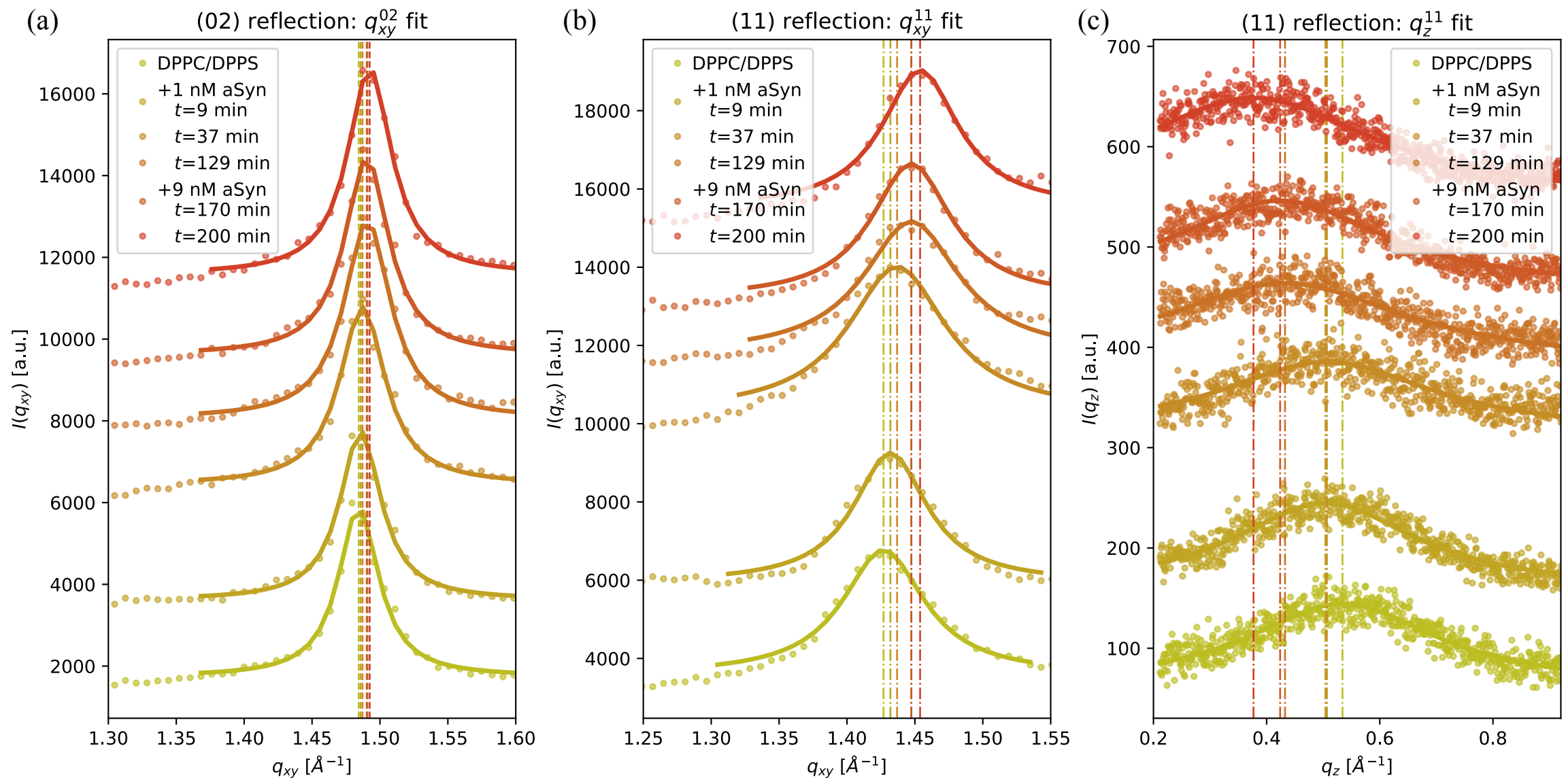

Fig. S8: GID fits corresponding to Fig. 3b, measured at constant monolayer area.

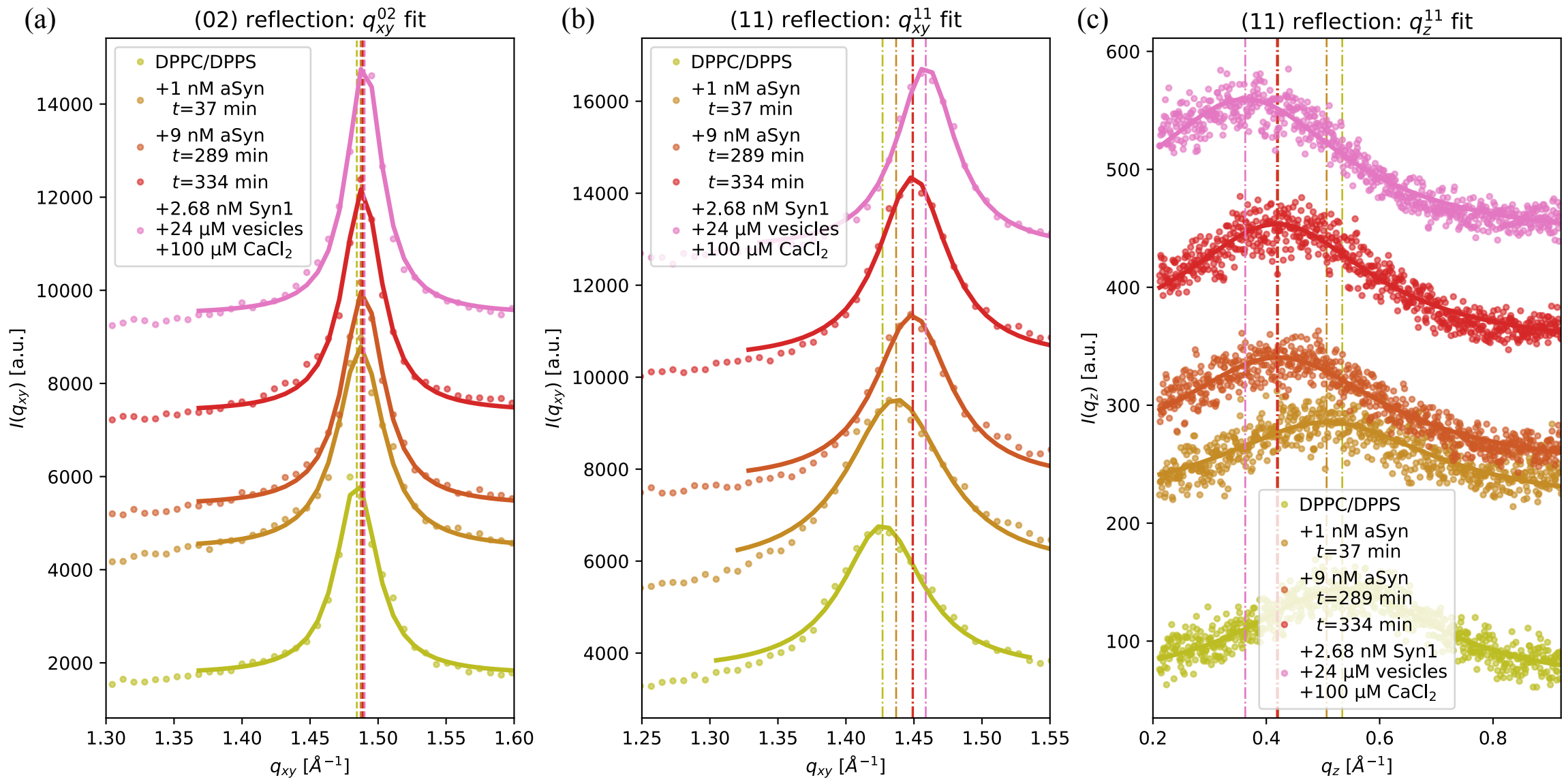

Fig. S9: GID fits corresponding to the A_c measurements near $\Pi = 28\,\mathrm{mN/m}$ shown in Fig. 3c

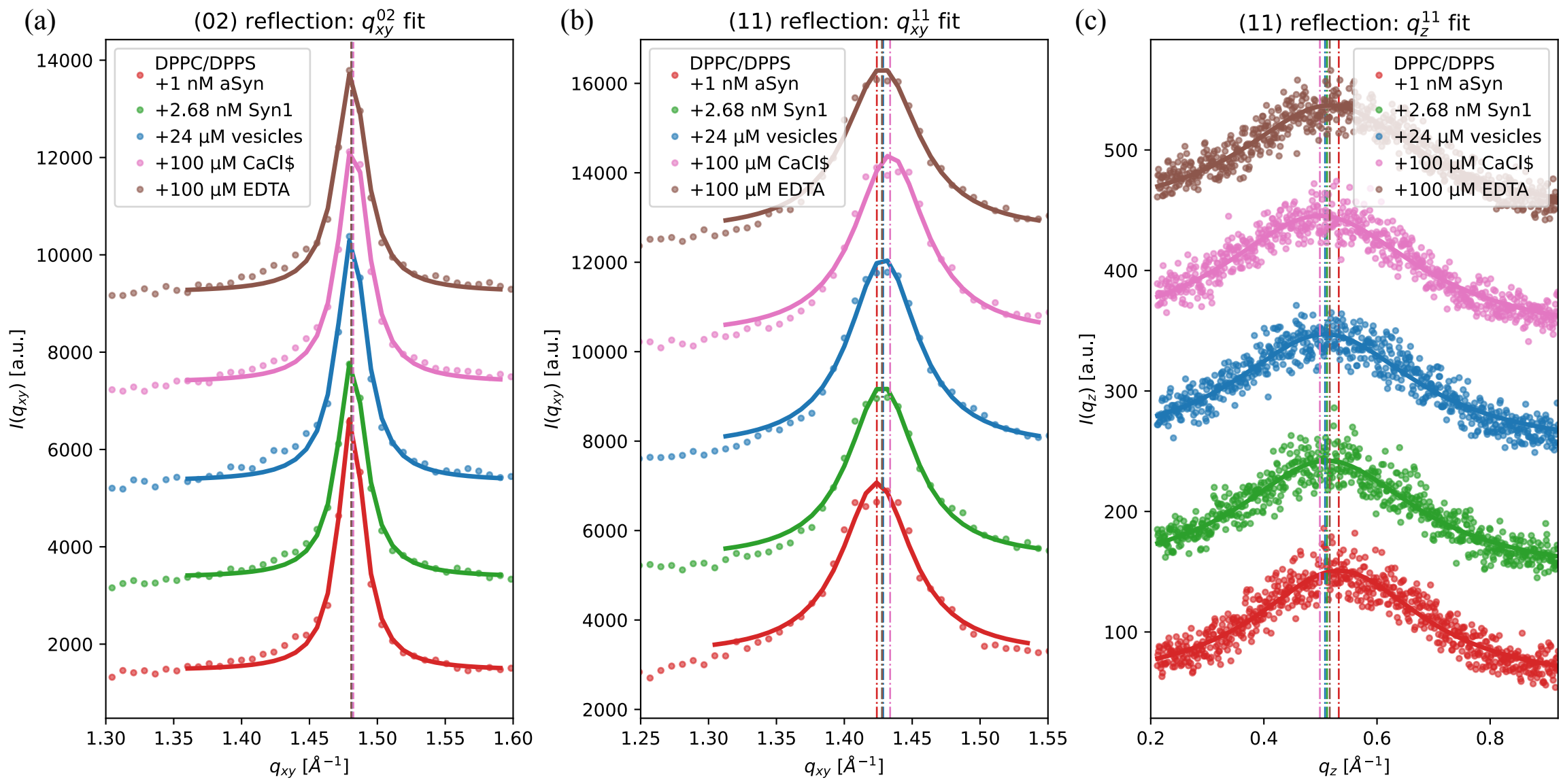

Fig. S10: GID fits corresponding to the A_c measurements at $\Pi = 20\,\mathrm{mN/m}$ shown in Fig. 3d

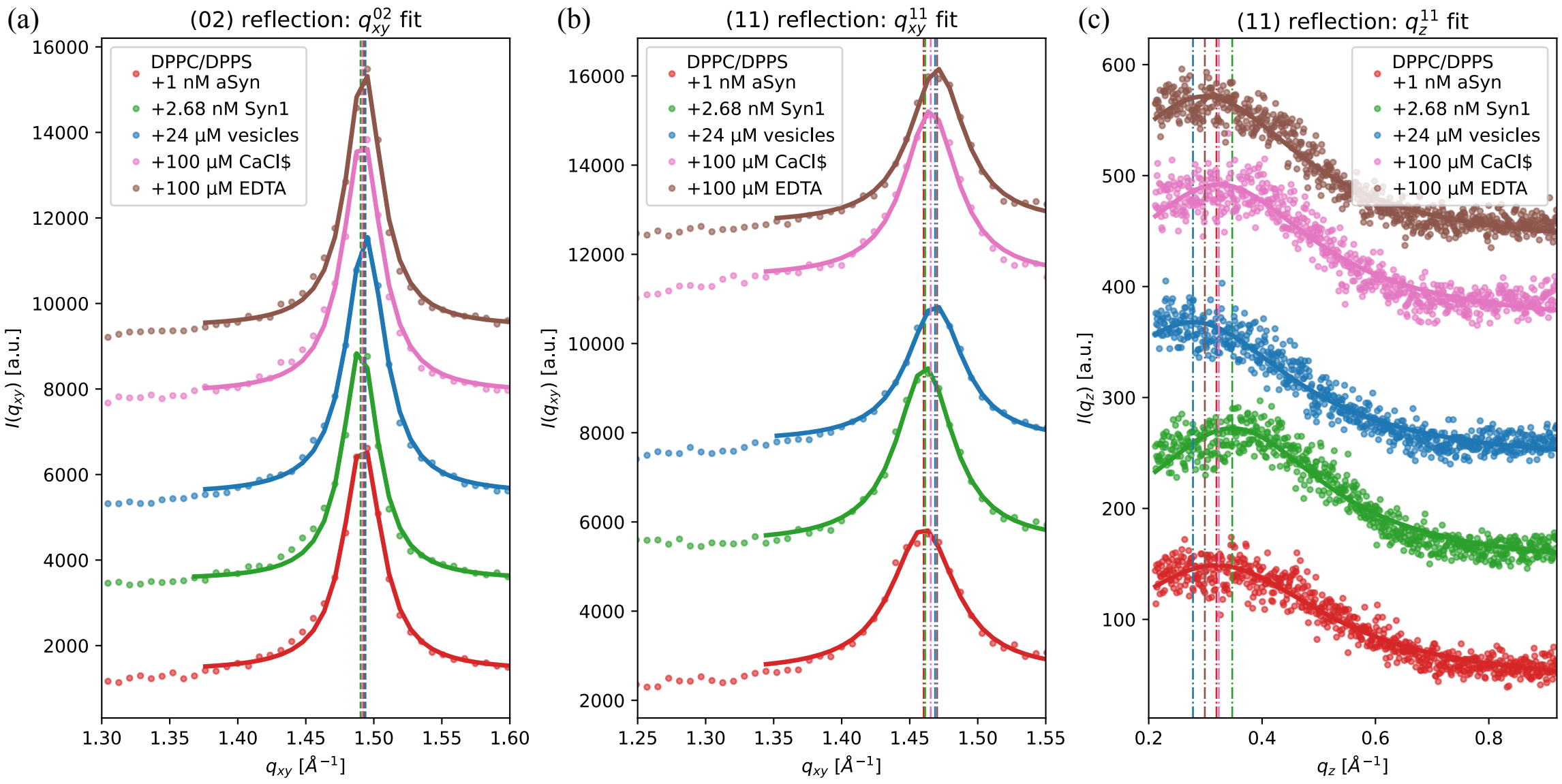

Fig. S11: GID fits corresponding to the A_c measurements at $\Pi = 30\,\mathrm{mN/m}$ shown in Fig. 3d

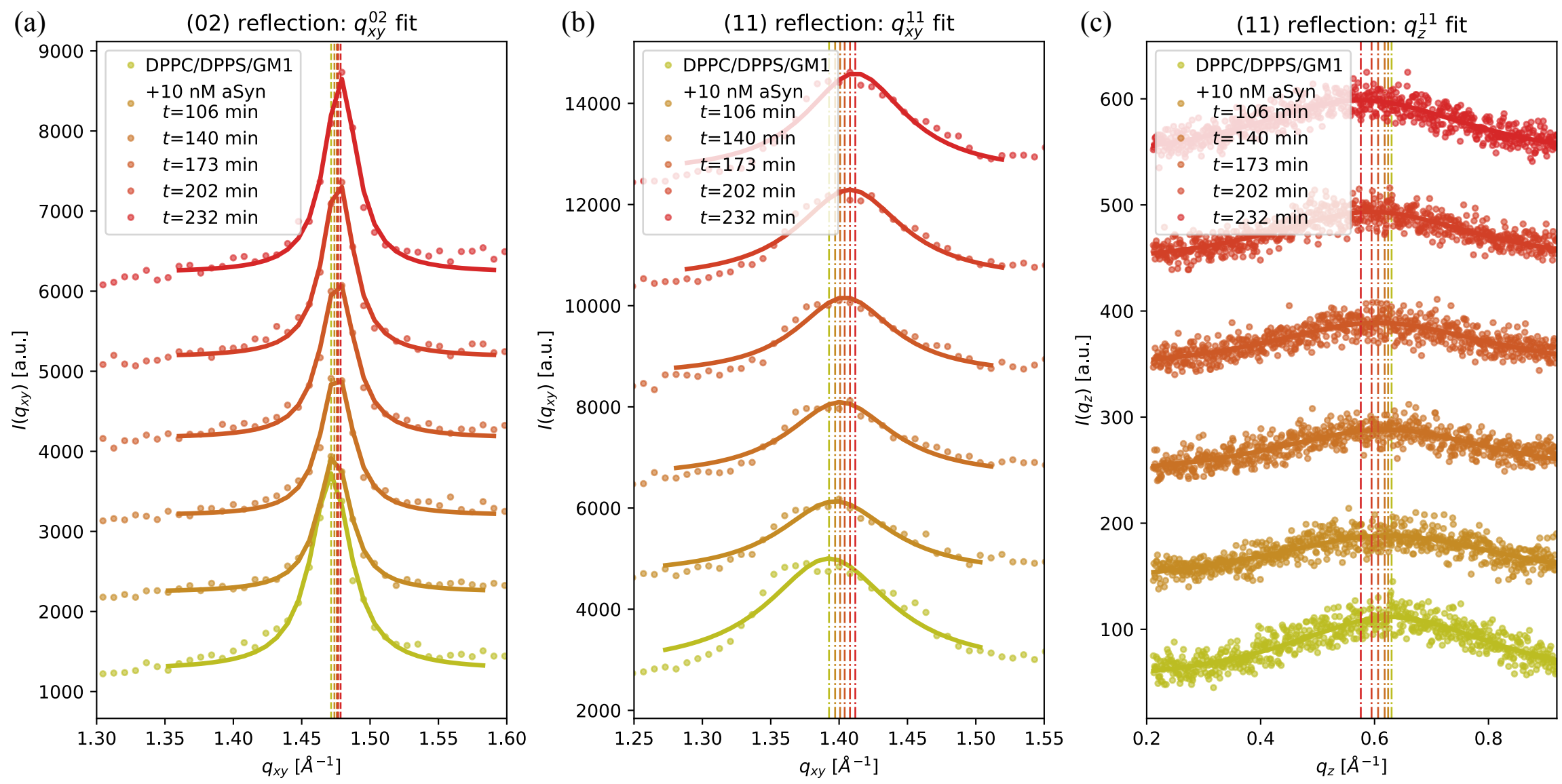

Fig. S12: GID fits for A_c measurements at constant area (starting at $\Pi_i = 20\,\mathrm{mN/m}$) shown in Fig. 3d

XRR fit parameters

	L_{tail} [Å]	ρ_{tail} [Å^{-3}]	$\sigma_{\text{tail-air}}$ [Å]	L_{head} [Å]	ρ_{head} [Å^{-3}]	$\sigma_{\text{head-tail}}$ [Å]	$\sigma_{\text{sub-head}}$ [Å]
DPPC/DPPS	16.45	0.2488	4.266	9.932	0.4881	4.829	2.755
+10 nM aSyn	16.6	0.2506	4.297	10.19	0.4914	4.769	2.844
+2.68 nM Syn1	16.66	0.2525	4.143	10.25	0.4799	4.517	2.823
+24 µM vesicles	18.23	0.2286	3.335	7.814	0.5807	5.761	4.078
+100 µM $CaCl_2$	16.5	0.2568	4.357	10.47	0.4795	4.473	2.679
+100 µM EDTA	19.86	0.2234	3.1	6.121	0.7052	6.551	4.667

Table S2: XRR fit parameters corresponding to Fig. 3e-f.

	L_{tail} [Å]	ρ_{tail} [Å^{-3}]	$\sigma_{\text{tail-air}}$ [Å]	L_{head} [Å]	ρ_{head} [Å^{-3}]	$\sigma_{\text{head-tail}}$ [Å]	L_{glycan} [Å]	ρ_{glycan} [Å^{-3}]	$\sigma_{\text{glycan-head}}$ [Å]	$\sigma_{\text{sub-glycan}}$ [Å]
DPPC/DPPS/GM1	16.65	0.2381	6.026	12.36	0.4709	4.437	11.83	0.3907	2.777	1.744
+10 nM aSyn, t=43 min	16.87	0.2335	5.967	12.12	0.4777	4.449	12.23	0.3912	2.882	1.722
t=316 min	16.74	0.201	6.386	12.71	0.4841	4.146	12.05	0.3919	2.723	1.443

Table S3: XRR fit parameters corresponding to Fig. 4c and 4e.

	L_{tail} [Å]	ρ_{tail} [Å^{-3}]	$\sigma_{\text{tail-air}}$ [Å]	L_{head} [Å]	ρ_{head} [Å^{-3}]	$\sigma_{\text{head-tail}}$ [Å]	L_{glycan} [Å]	ρ_{glycan} [Å^{-3}]	$\sigma_{\text{glycan-head}}$ [Å]	$\sigma_{\text{sub-glycan}}$ [Å]
DPPC/DPPS/GM1	16.79	0.2823	7.161	12.29	0.4716	4.15	12.97	0.3837	3.302	1.777
+10 nM aSyn, t=212 min	15.28	0.2808	9.227	12.2	0.4827	3.884	13.3	0.3791	2.57	2.037
t=241 min	16.27	0.2831	8.993	11.97	0.5052	4.171	13.82	0.3824	3.254	1.509

Table S4: XRR fit parameters corresponding to Fig. 4d and 4f.

	L_{tail} [Å]	ρ_{tail} [Å^{-3}]	$\sigma_{\text{tail-air}}$ [Å]	L_{head} [Å]	ρ_{head} [Å^{-3}]	$\sigma_{\text{head-tail}}$ [Å]	$\sigma_{\text{sub-head}}$ [Å]
DOPC/DOPS	12.92	0.3099	5.697	9.316	0.4343	3.114	2.557
+10 nM aSyn	12.67	0.3123	5.331	9.679	0.4311	3.144	2.333
+2.68 nM Syn1	12.57	0.3181	5.528	9.74	0.4247	2.745	2.321
+24 µM vesicles	12.31	0.3159	5.613	9.736	0.4219	2.885	2.183
+100 µM $CaCl_2$	13	0.3145	5.541	10.02	0.4251	2.957	2.301
+100 µM EDTA	13.02	0.2862	4.827	9.619	0.4391	3.578	2.584

Table S5: XRR fit parameters corresponding to Fig. 5a-b.

Langmuir trough control experiments

Surface pressure increase at constant monolayer area following aSyn injections

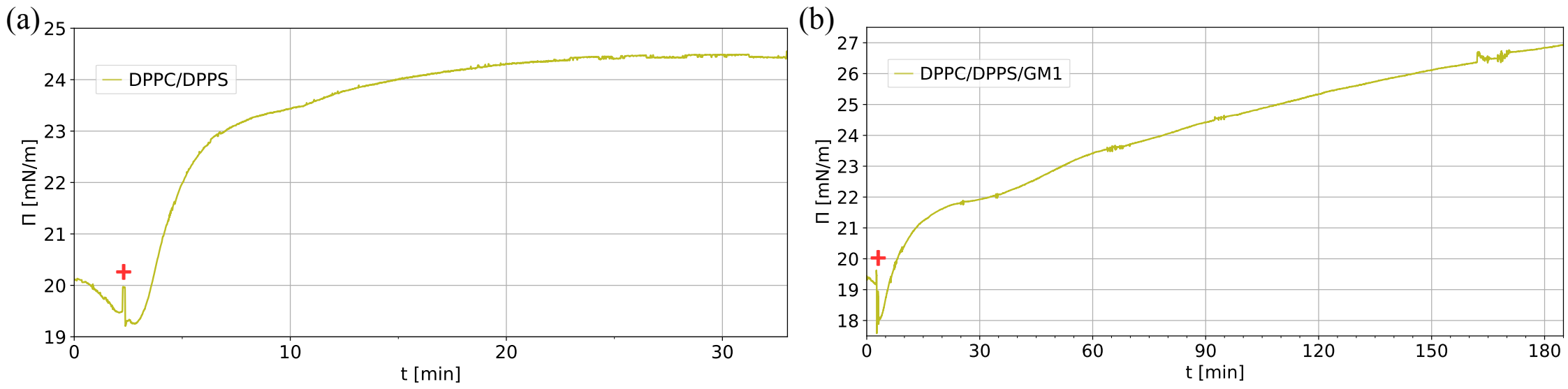

Fig. S13: Lipid monolayer pressure response to the injection of 10 nM aSyn (a) DPPC/DPPS (1:1) monolayer equilibrated near 20 mN/m. After injection of 10 nM aSyn (red +), the surface pressure increases by $\Delta\Pi \simeq 5$ mN/m within 30 min and finally saturates between 24.5 mN/m to 25 mN/m. (b) DPPC/DPPS/GM1 (2:1:1) monolayer equilibrated near 20 mN/m. After injection of 10 nM aSyn (red +), the surface pressure drops to $\Pi \simeq 18$ mN/m, followed by a steady increase of $\Delta\Pi \simeq 9$ mN/m over 180 min without reaching saturation.

Lipid domains in monolayers visualized by fluorescence microscopy

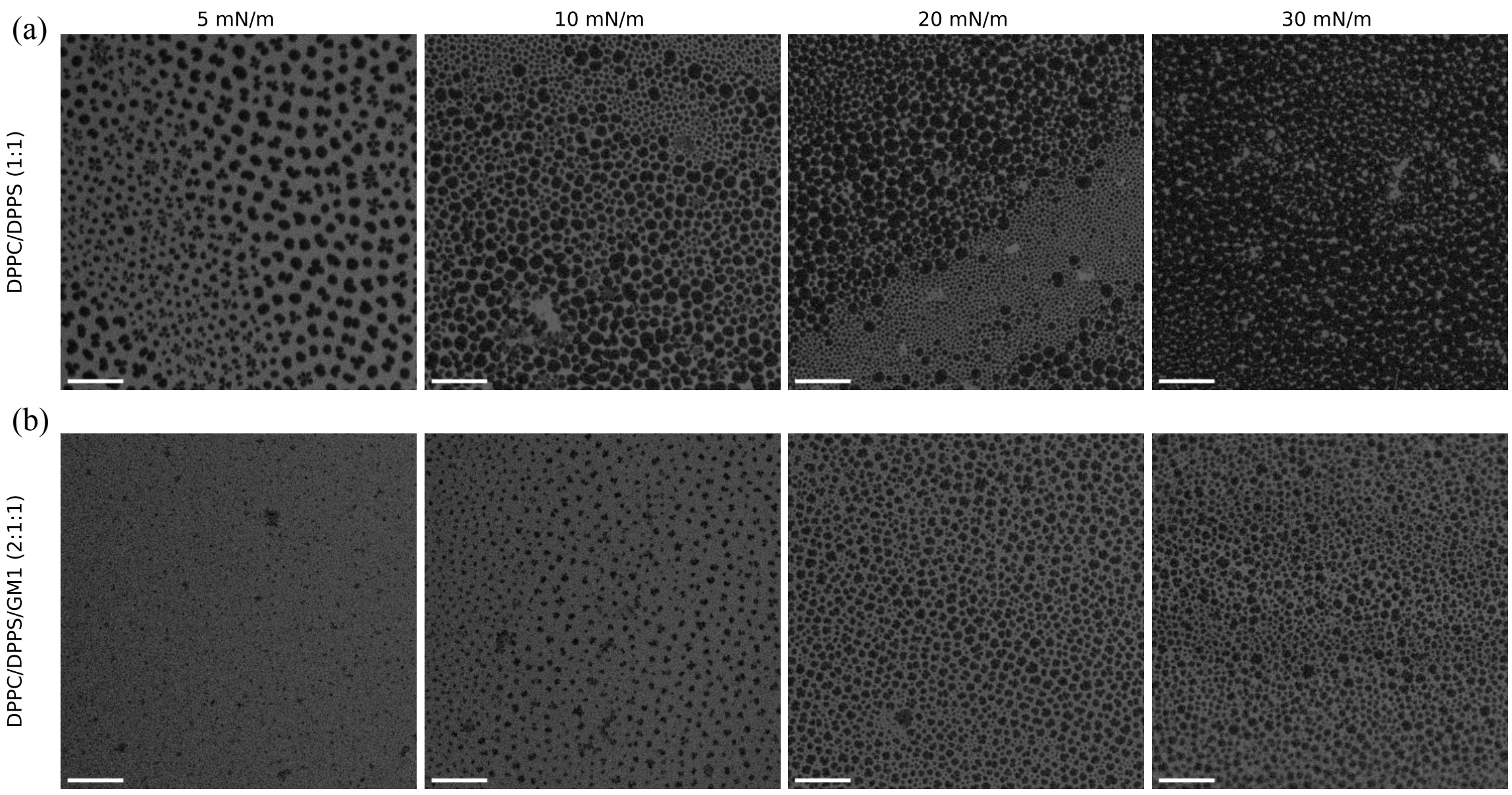

Fig. S14: Two-phase domain coexistence in lipid monolayers demonstrated by fluorescence microscopy. For these control experiments, monolayers containing a small amount (<2 mol%) of Texas Red DHPE were prepared in a smaller Langmuir trough ($5\times13\times0.4$ cm^3, ca. 26 ml) mounted below an Olympus BX61 microscope. Due to the preferential partitioning of the dye in the more disordered liquid-expanded (LE) phase [9], the LE phase appears bright (because the dye is present), while the condensed (LC) domains appear dark (because the dye is squeezed out). (a) DPPC/DPPS (1:1) monolayer. (b) DPPC/DPPS/GM1 (2:1:1) monolayer. Scale bar: 100 µm.

SAXS curves of vesicle-protein suspensions

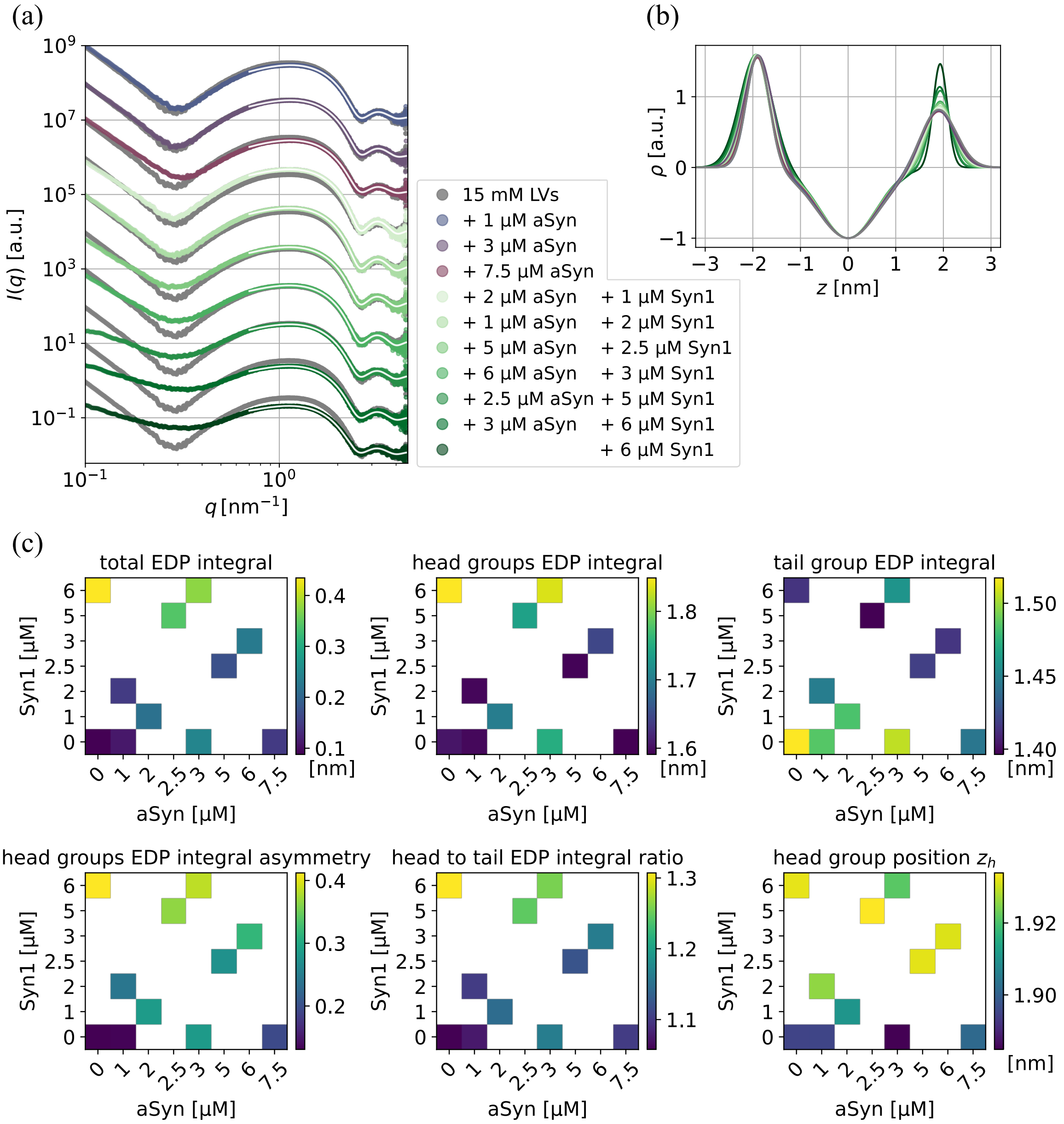

Fig. S15: Structure of LV suspensions with Syn1 and/or aSyn. (a) SAXS curves with least squares fits (white) in the region $q > 0.7\,\mathrm{nm}^{-1}$. The underlying fit parameters are shown in Table S10. The low q-regime is strongly influenced by the LV clustering (condensate formation) of Syn1 at concentrations above 3 µM. (b) EDPs corresponding to the fits. (c) Selected quantities of the flat asym. EDP model based on the fits in (a), analogous to Fig. 6b-c. At low µM concentrations, Syn1 has a larger effect on these parameters than aSyn.

SAXS results of LVs with aSyn variants

SAXS measurements were taken to compare two aSyn variants linked to familial forms of Parkinson's disease, A30P [10] and G14R [11], with wild type (WT) aSyn regarding their interactions with lipid vesicles. Both mutations are located within the lipid-binding N-terminus, implying a connection between lipid-binding properties and pathological aggregation. Samples containing 15 µM and 25 µM of the respective protein variant were measured and the resulting SAXS curves were fitted with the ellipsoidal asym. model (Fig. S16). Analogous to Fig. 6, certain interesting quantities were calculated from the EDP integrals $\Omega_{1,2,3}$ and are listed in Tab. S8.

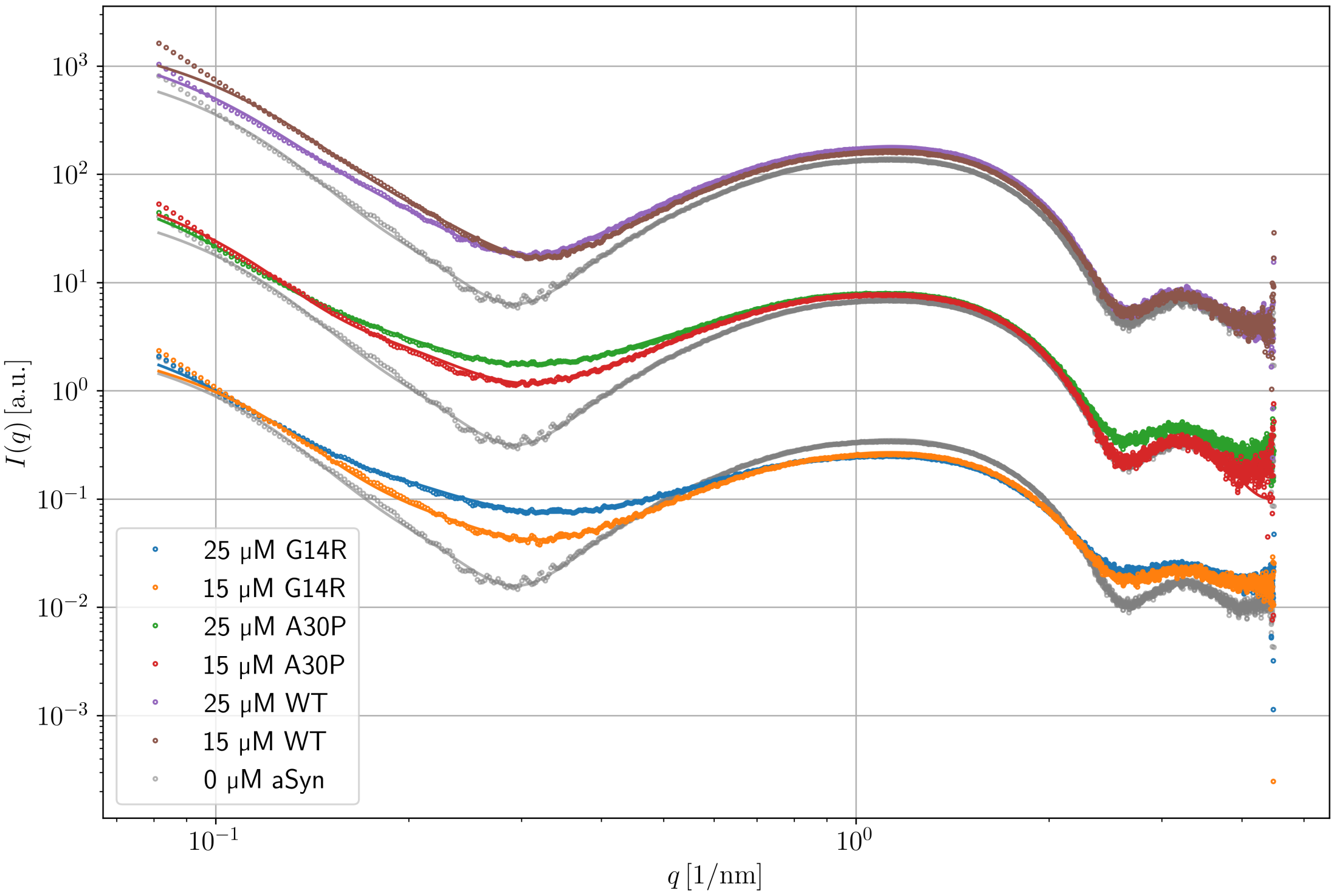

Fig. S16: Scattering curves and ellipsoidal asym. fits of LVs with different variants of aSyn, for clarity shown in three shifted groups of curves. For comparison, each group contains the 15 mM LV sample without aSyn. The 25 µM G14R aSyn and 25 µM A30P aSyn samples contained 13.24 mM LVs, all other samples contained 15 mM LVs. In general, the characterisitic minimum at ca. 0.3 nm^{-1} is filled up in the presence of proteins. The low-q SAXS signal of both variants differs slightly from that of WT aSyn.

Analysis of SAXS background

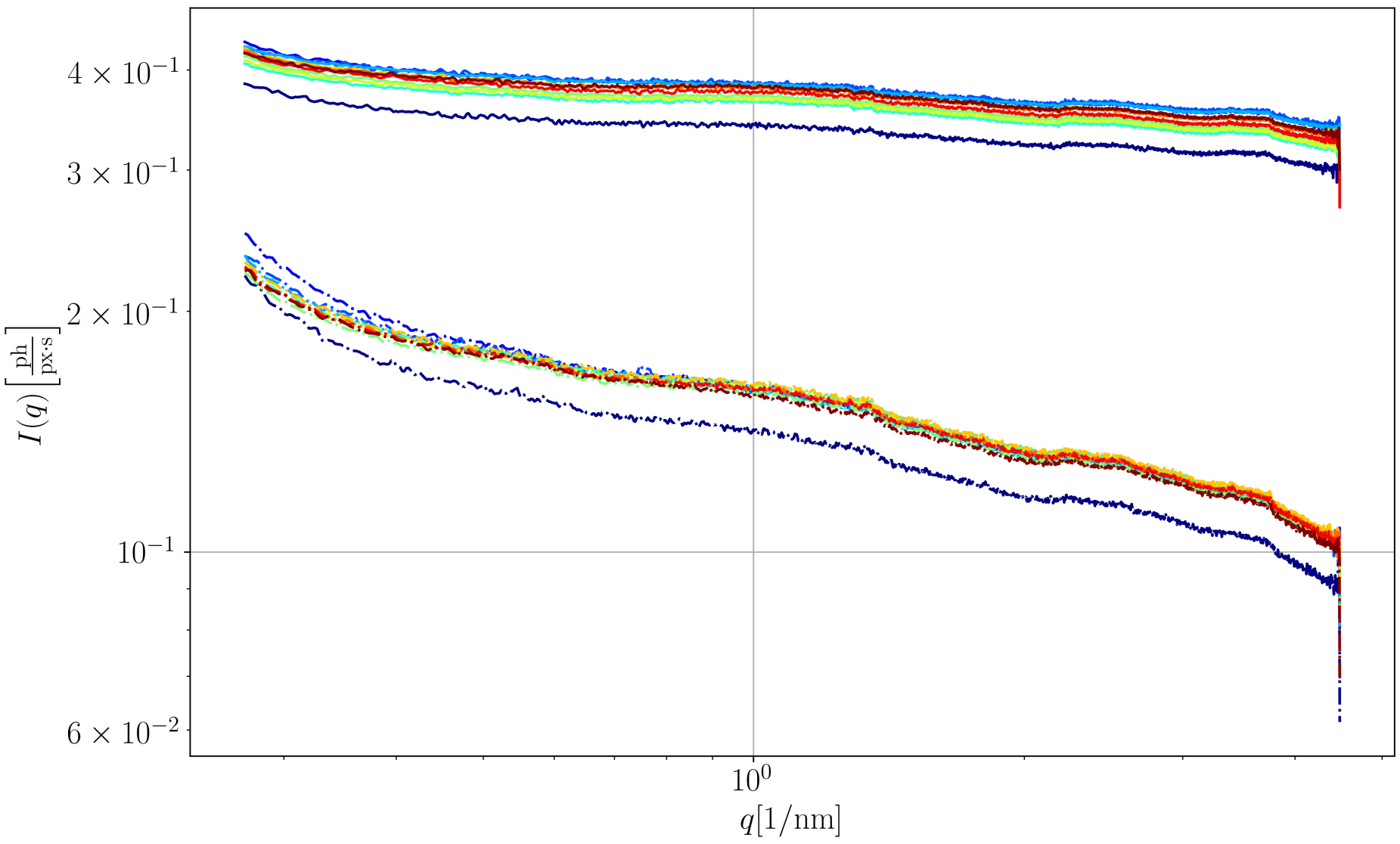

Fig. S17: The lower, dash-dotted group of curves shows the scattering in air only, while the upper group shows TBS curves which were measured shortly after a corresponding air curve of the same color. Since the vertical order of the air curves is only weakly correlated with that of the TBS curves, the background fluctuations are dominated by the different scattering properties of the capillaries. The fluctuations of the scattering in air only, mainly consequence of the beam current decay and the refilling of the synchrotron, are small in comparison. For clarity, the data is shown in the region $q > 0.27\,\mathrm{nm}^{-1}$.

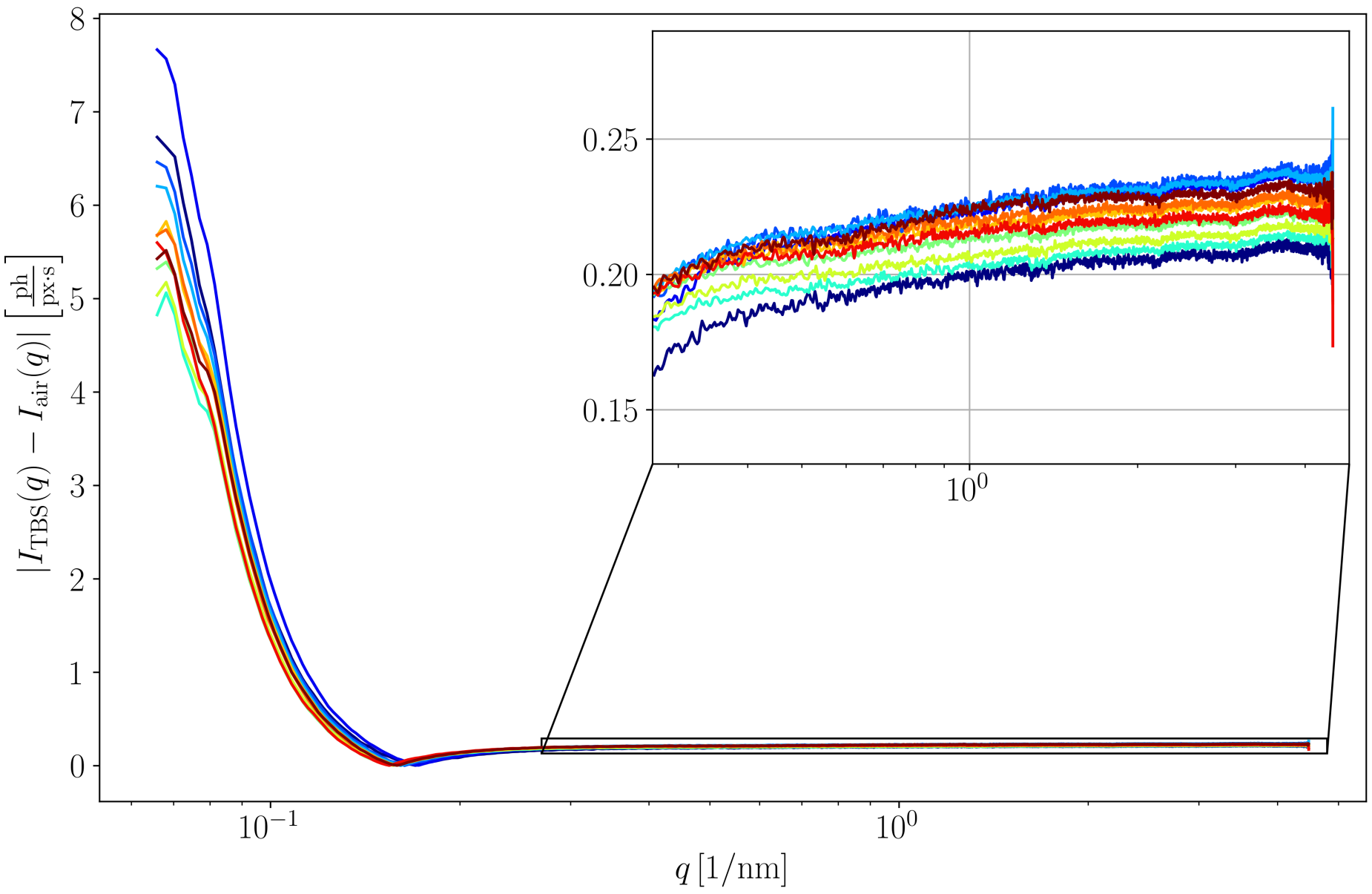

Fig. S18: Absolute differences of TBS and empty air scattering curves which were measured in close succession. The notable spread in the curves can be attributed to the large variance of capillary scattering.

SAXS fit parameters

	0 µM aSyn	0.3 µM aSyn	1 µM aSyn	3 µM aSyn	7.5 µM aSyn	9 µM aSyn	15 µM aSyn	25 µM aSyn
Total EDP integral $(\Omega_1 + \Omega_2 + \Omega_3)$ [nm]	0.328	0.327	0.331	0.331	0.394	0.312	0.421	0.368
Head group integral $(\Omega_1 + \Omega_3)$ [nm]	2.0	1.87	1.76	1.91	1.99	1.88	2.03	2.04
Tail group integral $\lvert\Omega_2\rvert$ [nm]	1.67	1.54	1.43	1.58	1.60	1.57	1.61	1.67
EDP asymmetry $\lvert\Omega_1 - \Omega_3\rvert/(\Omega_1 + \Omega_3)$	0.177	0.121	0.173	0.128	0.199	0.141	0.205	0.185
Ratio of head groups to tail groups $(\Omega_1 + \Omega_3)/\lvert\Omega_2\rvert$	1.20	1.21	1.23	1.21	1.25	1.20	1.26	1.22
z_h [nm]	1.75	1.80	1.84	1.84	1.77	1.88	1.83	1.85
R [nm]	10.0	9.82	9.3	10.27	10.62	11.16	10.47	10.37
σ_R [nm]	5.97	6.06	6.06	6.89	6.25	6.96	6.08	7.32
ε	1.13	1.16	1.13	1.08	1.17	1.02	1.07	1.01
σ_ε	0.103	0.159	0.112	0.237	0.217	0.212	0.25	0.219

Table S6: Comparison of selected quantities of ellipsoidal asym. fits to SAXS curves of aSyn+LVs, as shown in Fig. 6c. The lipid concentration was 15 mM in all samples. The underlying curves and fits are shown in Fig. 6a, the fitted parameters in Tab. S7.

	0 µM aSyn	0.3 µM aSyn	1 µM aSyn	3 µM aSyn	7.5 µM aSyn	9 µM aSyn	15 µM aSyn	25 µM aSyn
ρ_1	1.88	1.27	1.25	1.32	1.48	1.24	1.37	1.33
ρ_3	1.04	1.74	1.50	3.41	1.75	3.29	3.35	3.46
σ_1 [nm]	0.30	0.37	0.35	0.36	0.37	0.38	0.41	0.42
σ_3 [nm]	0.44	0.20	0.20	0.10	0.20	0.10	0.10	0.10
σ_2 [nm]	0.90	0.69	0.61	0.69	0.74	0.67	0.72	0.75
z_h [nm]	1.75	1.80	1.84	1.83	1.77	1.88	1.83	1.85
R [nm]	10.00	9.82	9.30	10.27	10.62	11.16	10.47	10.37
σ_R [nm]	5.97	6.06	6.06	6.89	6.25	6.96	6.08	7.32
ε	1.13	1.16	1.13	1.08	1.17	1.02	1.07	1.01
σ_ε	0.10	0.16	0.11	0.24	0.22	0.21	0.25	0.22
c_1	$6.3 \cdot 10^{-6}$	$2 \cdot 10^{-4}$	$2 \cdot 10^{-4}$	$6 \cdot 10^{-6}$	$5 \cdot 10^{-6}$	$8 \cdot 10^{-6}$	$8 \cdot 10^{-6}$	$8 \cdot 10^{-6}$
c_2 $[\frac{\mathrm{ph \cdot nm^{-c_3}}}{\mathrm{px \cdot s}}]$	0	0.2	0.29	0	0	0	0	0.001
c_3	0	0	0	0.01	0	0.01	0.01	0.15
c_4 $[\frac{\mathrm{ph}}{\mathrm{px \cdot s}}]$	0.009	0.08	0.004	0.006	0.008	0.009	0.007	0.005
$\chi^2_{\mathrm{red,Poisson}}$	23.48	19.4	23.0	14.4	12.1	15.8	31.6	13.7
$\chi^2_{\mathrm{red,operative}}$	6.09	2.3	2.8	2.7	2.8	2.5	3.6	3.4

Table S7: Fit results of ellipsoidal asym. fits of aSyn-LV scattering curves, which are shown in Fig. 6. The lipid concentration was 15 mM in all samples.

	0 µM aSyn	15 µM WT	25 µM WT	15 µM A30P	25 µM A30P	15 µM G14R	25 µM G14R
Total EDP integral $(\Omega_1+\Omega_2+\Omega_3)$ [nm]	0.328	0.421	0.368	0.322	0.271	0.344	0.368
Head group integral $(\Omega_1+\Omega_3)$ [nm]	2.0	2.03	2.04	2.17	1.84	2.03	2.34
Tail group integral $\lvert\Omega_2\rvert$ [nm]	1.67	1.61	1.67	1.85	1.57	1.68	1.97
EDP asymmetry $\lvert\Omega_1-\Omega_3\rvert/(\Omega_1+\Omega_3)$	0.177	0.205	0.185	0.00705	0.0926	0.0214	0.0572
Ratio of head groups to tail groups $(\Omega_1+\Omega_3)/\lvert\Omega_2\rvert$	1.2	1.26	1.22	1.17	1.17	1.20	1.19
z_h [nm]	1.75	1.83	1.85	1.76	1.83	1.78	1.78
R [nm]	10.0	10.5	10.4	9.91	8.04	8.00	9.12
σ_R [nm]	5.97	6.08	7.32	6.20	7.51	5.85	6.72
ε	1.13	1.07	1.01	1.10	1.11	1.13	0.912
σ_ε	0.103	0.25	0.219	0.335	0.277	0.226	0.462

Table S8: Comparison of selected quantities of ellipsoidal asym. fits to SAXS curves of aSyn+LVs, analogous to Fig. 6b-c. The focus lies here on the aSyn variants G14R and A30P. The underlying curves and fits are shown in Fig. S16.

	0 µM aSyn	15 µM WT	25 µM WT	15 µM A30P	25 µM A30P	15 µM G14R	25 µM G14R
ρ_1	1.88	1.37	1.33	4.00	3.40	1.90	5.06
ρ_3	1.04	3.35	3.46	1.48	1.52	1.31	1.82
σ_1 [nm]	0.30	0.41	0.42	0.12	0.10	0.25	0.10
σ_3 [nm]	0.44	0.10	0.10	0.37	0.28	0.37	0.35
σ_2 [nm]	0.90	0.72	0.75	0.91	0.66	0.81	1.00
z_h [nm]	1.75	1.83	1.85	1.76	1.83	1.78	1.78
R [nm]	10.00	10.47	10.37	9.91	8.04	8.00	9.12
σ_R [nm]	5.97	6.08	7.32	6.20	7.51	5.85	6.72
ε	1.13	1.07	1.01	1.10	1.11	1.13	0.91
σ_ε	0.10	0.25	0.22	0.34	0.28	0.23	0.46
c_1	$6.3\cdot10^{-6}$	$8\cdot10^{-6}$	$8\cdot10^{-6}$	$6\cdot10^{-6}$	$9\cdot10^{-6}$	$6\cdot10^{-6}$	$5\cdot10^{-6}$
c_2 $[\frac{\text{ph}\cdot\text{nm}^{-c_3}}{\text{px}\cdot\text{s}}]$	0	0.00	0.001	0	0	0	0
c_3	0	0.01	0.15	0	0	0	0
c_4 $[\frac{\text{ph}}{\text{px}\cdot\text{s}}]$	0.009	0.007	0.005	0.003	0.01	0.015	0.013
$\chi^2_{\text{red,Poisson}}$	23.48	31.6	13.7	8.4	4.6	14.2	3.3
$\chi^2_{\text{red,operative}}$	6.09	3.6	3.4	13.9	3.7	2.5	2.6

Table S9: Fit results of ellipsoidal asym. fits of aSyn-LV scattering curves, which are shown in Fig. S16. The focus here lies on the aSyn variants G14R and A30P.

Syn1 [µM]	6	6	5	3	2.5	2	1	0	0	0	0
aSyn [µM]	0	3	2.5	6	5	1	2	7.5	3	1	0
ρ_1	1.58	1.60	1.60	1.60	1.60	1.60	1.60	1.56	1.60	1.60	1.60
ρ_3	1.47	1.16	1.07	0.939	0.913	0.876	0.870	0.800	0.822	0.826	0.834
σ_1 [nm]	0.341	0.332	0.310	0.278	0.263	0.259	0.291	0.251	0.297	0.234	0.237
σ_3 [nm]	0.150	0.201	0.211	0.248	0.263	0.290	0.299	0.346	0.336	0.367	0.370
σ_2 [nm]	0.590	0.613	0.581	0.586	0.589	0.606	0.632	0.610	0.653	0.634	0.653
z_h [nm]	1.93	1.92	1.93	1.93	1.93	1.92	1.91	1.90	1.88	1.90	1.89
c_1	0.303	0.359	0.512	0.610	0.667	0.743	0.756	0.502	0.536	0.520	0.592
c_4 $[\frac{\text{ph}}{\text{px}\cdot\text{s}}]$	0.00882	0.00704	0.0178	0.0085	0.0111	0.0112	0.0145	0.0104	0.00983	0.0103	0.0095

Table S10: Fit results of flat asym. fits of aSyn-Syn1-LV scattering curves, which are shown in Fig. 8a and in Fig. S15. The EDPs are shown in Fig. 8b and S15b.

References

[1] H. Bruns, T. Czajka, C. Neuhaus, M. Jankowski, D. Pontoni, C. Hoffmann, D. Milovanovic, T. Salditt, Synapsin shields a lipid monolayer from interactions with vesicles and associated structural rearrangements. Biophysical Journal (2026). https://doi.org/10.1016/j.bpj.2025.12.004

[2] V.M. Kaganer, H. Möhwald, P. Dutta, Structure and phase transitions in langmuir monolayers. Reviews of Modern Physics **71**(3), 779 (1999)

[3] D. Marsh, Lateral order in gel, subgel and crystalline phases of lipid membranes: Wide-angle x-ray scattering. Chemistry and physics of lipids **165**(1), 59–76 (2012)

[4] M.R. Brzustowicz, A.T. Brunger, X-ray scattering from unilamellar lipid vesicles. Journal of Applied Crystallography **38**(1), 126–131 (2005). https://doi.org/10.1107/S0021889804029206

[5] C. Neuhaus, M.L. Stammer, J. Alfken, M. Osterhoff, K. Komorowski, A. Major, J. Bielecki, S. Rafie-Zinedine, R. de Wijn, R. Letrun, J. E, R. Bean, T. Salditt, Structure and polydispersity of single lipid vesicles by small-angle x-ray scattering at european xfel. Communications Physics (2026). https://doi.org/10.1038/s42005-026-02551-5

[6] C. Hoffmann, R. Sansevrino, G. Morabito, C. Logan, R.M. Vabulas, A. Ulusoy, M. Ganzella, D. Milovanovic, Synapsin condensates recruit alpha-synuclein. Journal of Molecular Biology **433**(12), 166961 (2021). https://doi.org/10.1016/j.jmb.2021.166961

[7] J. Alfken, C. Neuhaus, A. Major, A. Taskina, C. Hoffmann, M. Ganzella, A. Petrovic, D. Zwicker, R. Fernández-Busnadiego, R. Jahn, D. Milovanovic, T. Salditt, Vesicle condensation induced by synapsin: condensate size, geometry, and vesicle shape deformations. European Physical Journal E **47**, 8 (2024). https://doi.org/10.1140/epje/s10189-023-00404-5

[8] S. Maltseva, D. Kerr, M. Turke, E.J. Adams, K.Y. C., Parkinson's disease-associated mutations in a-synuclein alters its lipid-bound state. Biophysical Journal **123**(12), 1610–1619 (2024)

[9] H.M. McConnell, Structures and transitions in lipid monolayers at the air-water interface. Annual Review of Physical Chemistry **42**(1), 171–195 (1991)

[10] R. Krüger, W. Kuhn, T. Müller, D. Woitalla, M. Graeber, S. Kösel, H. Przuntek, J.T. Epplen, L. Schols, O. Riess, Alasopro mutation in the gene encoding α-synuclein in parkinson's disease. Nature genetics **18**(2), 106–108 (1998)

[11] C. Brücke, M. Al-Azzani, N. Ramalingam, M. Ramón, R.L. Sousa, F. Buratti, M. Zech, K. Sicking, L. Amaral, E. Gelpi, et al., A novel alpha-synuclein g14r missense variant is associated with atypical neuropathological features. Molecular neurodegeneration **20**(1), 98 (2025)
